# The Limited Evidence Base for Multilevel Lumbar Interbody Fusion and Its Consequences for Clinical Conclusions: A Systematic Review

**DOI:** 10.3390/jcm15062289

**Published:** 2026-03-17

**Authors:** Evan R. Simpson, Casey Slattery, Kalyn Smith, Jesse Caballero, Michael Gordon, Gerald Alexander, Jon White, Jeffrey Deckey, Jeremy Smith, Vance Gardner

**Affiliations:** 1 Hoag Orthopedics, Irvine, CA 92618, USA; 2 Hoag Orthopedic Institute, Irvine, CA 92618, USA

**Keywords:** multilevel, lumbar interbody fusion, PLIF, OLIF, TLIF, LLIF

## Abstract

**Background/Objectives**: Lumbar interbody fusion (LIF) is widely utilized to treat multilevel degenerative lumbar spine pathologies. This systematic review aimed to comprehensively review lateral and posterior multilevel LIF procedures and their clinical and radiographic outcomes. **Methods**: Following the PRISMA guidelines, a search of PubMed, Embase, Web of Science, and Cochrane identified eligible studies. Patient demographics, as well as clinical and radiographic outcomes were collected. Risk of bias was assessed using the MINORS criteria, while randomized trials were evaluated using the RoB-2 tool. An extensive subgroup analysis was completed when that was possible. **Results**: A total of 45 studies were included consisting of 5623 patients. The pooled outcomes indicated that TLIF demonstrated the lowest operative duration (198.7 ± 77.83 min) and LOS (5.09 ± 2.5 days), alongside favorable ODI (33.68 ± 6.43), VAS leg pain (5.39 ± 0.66), and VAS back pain (4.67 ± 0.79) score gains. Comparative evidence found that LLIF and OLIF provided advantageous radiographic improvement to the posterior approaches. Comparative evidence on techniques challenged the use of autogenous bone within PLIF, PEEK over HA/PA66 cages, and found no advantages in unilateral decompression within TLIF. There was minimal clinical difference in evidence assessing MIS (minimally invasive) vs. open-TLIF or unilateral vs. bilateral pedicle screw fixation (PSF). **Conclusions**: This is the first systematic review of the multilevel LIF literature, revealing that while pooled data favored TLIF, a publication bias was detected, and comparative evidence reported advantages for lateral and oblique approaches. Given the lack of conclusive evidence, robust study designs are needed to guide clinical decision-making for multilevel lumbar pathology.

## 1. Introduction

As a result of documented positive clinical outcome and advancements, the number of spinal fusion procedures has been steadily increasing within the United States [1,2,3]. Further evidenced by a 170.9% increase from 1998 to 2008, the incidence of lumbar fusion procedures grew at an accelerated rate [4]. Lumbar interbody fusion (LIF) specifically has seen increases from 13.6% to 32%, whereas other fusion options, such as posterolateral fusion (PLF), have decreased [5,6,7,8].

Lateral procedures, such as lateral lumbar interbody fusion (LLIF), extreme lateral interbody fusion (XLIF, NuVasive, San Diego, CA, USA), and direct lateral interbody fusion (DLIF, Medtronic, Minneapolis, MN, USA), facilitate sagittal and coronal deformity correction through a lateral retroperitoneal transpsoas approach [9,10,11,12]. Through direct visualization, posterior lumbar interbody fusion (PLIF) can achieve decompression and restoration of interbody height with a single incision but can cause injury due to the retraction of muscles and nerves [10,13,14]. While transforaminal lumbar interbody fusion (TLIF) offers advantages such as reduced risk of injury and bilateral anterior column support via a single unilateral incision, lordosis restoration remains limited [15,16,17]. Oblique lateral interbody fusion (OLIF) proceeds anterior to the psoas but presents similar possibilities of vascular injury seen in the anterior approach [10,18,19,20,21]. While providing an array of tools for the surgeon, an informed consideration of benefits and risks for each LIF procedure is paramount. This is especially true in multilevel procedures, as revision risk increases with greater instrumentation length [22].

While consensus on a superior procedure remains in question, previous reviews have demonstrated the clinical success of varying LIF approaches [23,24,25]. This is limited to single-level procedures, and, to the authors’ knowledge, there is no prior study that has extensively reported on exclusively multilevel LIF procedures. Thus, this systematic review aims to thoroughly present all radiographic and clinical outcomes of lateral and posterior multilevel lumbar interbody fusion in the current literature.

## 2. Materials and Methods

### 2.1. Search Strategy and Eligibility Criteria

This systematic review was designed in compliance with the Preferred Reporting Items for Systematic Reviews and Meta-analyses (PRISMA) guidelines [26] and was not prospectively registered in the International Prospective Register of Systematic reviews (PRISMA 2020 Checklist). A literature search was conducted by two authors using PubMed, Embase, Web of Science, and Cochrane Central Register of Controlled Trials (CENTRAL) from database inception to present. Any conflicts were resolved by the senior author. The literature search can be found in Table S1. Inclusion criteria consisted of level 1–4 studies evaluating outcomes of lateral and posterior LIF of two or more levels, follow-up of at least 1 year, one or more patient-reported outcome measures (PROM), radiographic measurements, postoperative complications, and fusion rates. Studies using LIF with posterior instrumentation were included. Exclusion criteria included any non-full-text articles, biomechanical or cadaver studies, editorial commentaries, review articles, studies reporting fusion for infection or tumor, studies reporting on the use of an additional procedure such as PLF, posterior, or three column osteotomy, and traumatic lumbar spondylolisthesis as defined by Konan et al. [5].

### 2.2. Data Extraction

The data was extracted from the included studies by two authors and transferred to a Microsoft Excel (Microsoft Corp, Redmond, WA, USA) spreadsheet. The following data was extracted from each study: author name, publication year, level of evidence, demographics, operative details, radiographic outcomes, clinical outcomes, complications, conclusions, and limitations. Complications were delineated as revisions/reoperations, dural tear, adjacent segment disease/degeneration (ASD), symptomatic hematoma, myocardial infarction, psoas weakness, delayed wound healing, deep wound infection, superficial wound infection, pseudoarthrosis, rod fracture/hardware failure, pulmonary embolism, deep vein thrombosis, neurological complications, adjacent disk herniation, urinary tract infection, and all-cause complications not previously listed. All data was reported as mean with standard deviation and range, when possible. When data was presented without a standard deviation, the previously published approaches were used to derive an estimate [27,28]. All radiographic outcomes and PROMs were reported as change values between preoperative and postoperative measurements to assess the effect of the intervention. Due to significant heterogeneity in study design, a meta-analysis was not performed.

### 2.3. Quality Assessment

Each article underwent independent bias assessment by two authors. Non-randomized studies were assessed using the methodological index for non-randomized studies (MINORS) criteria [29]. Higher scores, indicating reduced bias, allow non-comparative studies to score up to 16 points and comparative studies up to 24 points. Non-comparative study scores were assessed as 0–4 (very low-quality evidence), 5–7 (low-quality evidence), 8–12 (fair-quality evidence), and greater than 13 (high-quality evidence). Comparative study scores were assessed as 0–6 (very low-quality evidence), 7–10 (low-quality evidence), 11–15 (fair-quality evidence), 16–20 (good-quality evidence), and greater than 21 (high-quality evidence). Randomized studies were assessed using the Cochrane risk-of-bias tool for randomized controlled trials (RoB-2) [30].

## 3. Results

### 3.1. Study Selection

**Figure 1:**
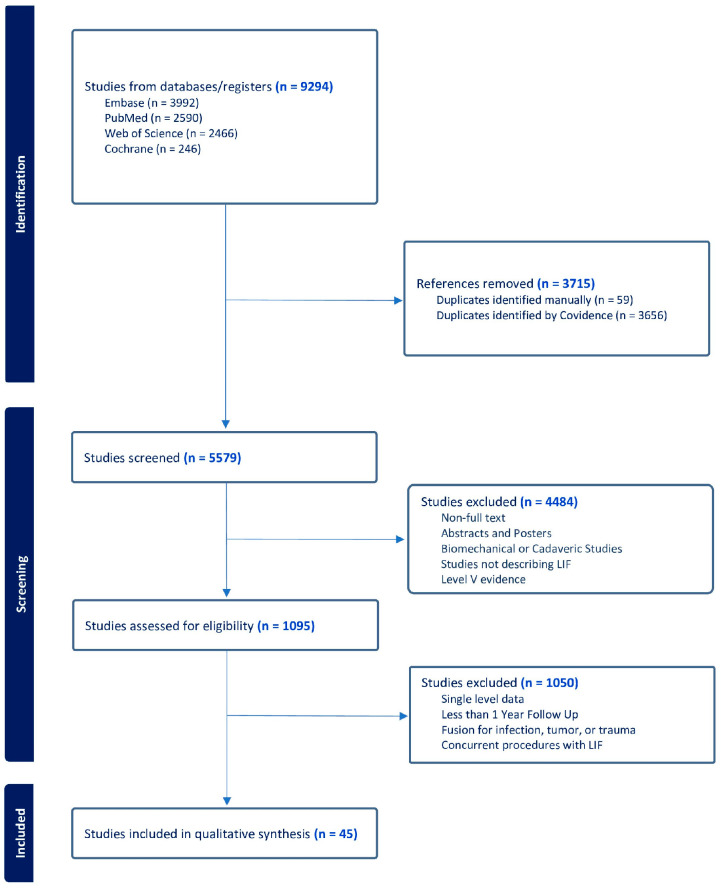
Preferred Reporting Items for Systematic Reviews and Meta-Analyses (PRISMA) flowchart of study selection.

A comprehensive search of all available lateral and posterior multilevel LIF literature was performed. The query of online databases identified 9294 studies. After initial screening, 1095 full texts were assessed for eligibility, leading to an inclusion of 45 studies (Figure 1).

### 3.2. Study Characteristics

Of the 45 included studies, two were randomized-controlled-trials (RCT) [31,32], three were prospective cohort studies [33,34,35], 19 were retrospective cohort studies [36,37,38,39,40,41,42,43,44,45,46,47,48,49,50,51,52,53,54], and 21 were case series [55,56,57,58,59,60,61,62,63,64,65,66,67,68,69,70,71,72,73,74,75]. A total of 25 studies reported on TLIF [32,33,34,35,36,37,38,41,42,43,44,52,53,54,58,61,62,63,64,67,68,69,72,73,74], 17 studies reported on PLIF [31,39,40,45,46,47,48,49,51,56,57,59,60,66,70,71,75], four studies reported on LLIF [36,46,55,65], and two studies reported on OLIF [50,52]. With 5623 total patients included, 4318 patients underwent TLIF, 1058 patients underwent PLIF, 110 patients underwent LLIF, and 137 patients underwent OLIF. PLIF reported the longest mean follow-up (48.80 ± 26.74 months), and TLIF followed (33.18 ± 5.67 months), while LLIF and OLIF had comparable shorter follow-up durations (21.40 ± 4.86 and 21.39 ± 12.47 months, respectively). Patients who underwent LLIF (68.63 ± 6.67 years) were slightly older compared to the other LIF cohorts, where mean ages were 61.21 ± 8.24 (PLIF), 61.22 ± 9.77 (TLIF), and 64.68 ± 10.6 (OLIF) years. The number of fused levels was relatively consistent across TLIF and PLIF (2.13 ± 0.33 and 2.13 ± 0.22, respectively), increasing slightly for LLIF (2.27 ± 0.25) and OLIF (2.64 ± 0.6). A detailed summary of reported study and patient characteristics can be found in Table 1. All individual study indications for surgery, surgical details, author conclusions, and limitations can be found in Table S2.

### 3.3. Pooled Outcomes

#### 3.3.1. Operative Details

The majority of studies reported using PSF to augment the LIF procedure; two studies were exceptions [40,55]. PSF varied significantly with unilateral/bilateral and open/percutaneous placement (Table S2). Similarly, studies used autograft or allograft, with or without the use of bone morphogenic protein (BMP) to aid in arthrodesis. When weighted pooled mean values were analyzed, TLIF demonstrated the shortest operative duration at 198.7 ± 77.83 min and the shortest LOS at 5.09 ± 2.5 days. The LLIF procedure was associated with the lowest reported intraoperative blood loss of 229 ± 125.6 mL despite this value reflecting the total time of the combined LLIF and posterior instrumentation (Table 2).

#### 3.3.2. Patient-Reported Outcome Measures

Three PROMs were reported by a large margin: Oswestry disability index (ODI), visual analog scale (VAS) leg pain, and VAS back pain. The collected TLIF data reported the greatest score improvement across ODI (33.68 ± 6.43), VAS leg pain (5.39 ± 0.66), and VAS back pain (4.67 ± 0.79). A summary of collected PROMs can be found in Table 3; however, no OLIF PROM data was available.

#### 3.3.3. Radiographic Outcomes

Reviewing the pooled radiographic outcome data, the TLIF procedure had the greatest reported changes in thoracic kyphosis (TK) (3.56 ± 3.78°) and sacral slope (SS) (4.67 ± 2.55°). The PLIF procedure had the largest reported changes in pelvic incidence (PI) (1.79 ± 1.4°). The LLIF procedure had the largest reported changes in segmental lordosis (SL) (4.3 ± 1.2°), pelvic tilt (PT) (3.95 ± 0.41°), and pelvic incidence–lumbar lordosis (PI–LL) mismatch (8.01 ± 3.09°). The OLIF procedure had the greatest reported fusion rate (96.7%), as well as the largest lumbar lordosis (LL) (8.1 ± 2.4°) and sagittal vertical axis (SVA) (30.4 ± 32.2°) change; however, only a single OLIF study reported radiographic outcomes (Table 4). The data on the pooled disk height (DH) or foraminal height (FH) restoration was not available by procedure.

#### 3.3.4. Complications

To facilitate comparison, complications were stratified and reported as both total event count and weighted rate (Table S3). From the pooled complication data, the LLIF procedure had the greatest weighted rate of complications (71.11%) and, the PLIF procedure had the lowest weighted rate of complications (13.91%); however the LLIF procedure had substantially less data (patient N = 45). TLIF had a weighted complication rate of 24.83%, and OLIF had a weighted complication rate of 15.33%. All complications in the “Other” category are fully listed with their individual N in Table S4.

### 3.4. Comparative Outcomes

#### 3.4.1. Procedural Comparison

Chong et al. [36] assessed two-level MIS LLIF and MIS TLIF, finding no differences in operative duration, LOS, or 2-year PROM improvement. The authors found LLIF to be superior for blood loss (229.0 ± 125.6 vs. 302.4 ± 97.1 mL), DH restoration at L3–L4 (4.1 ± 2.4 vs. 1.2 ± 1.9 mm), DH restoration at L4–L5 (3.0 ± 3.5 vs. −0.1 ± 4.4 mm), FH restoration at L3–L4 (3.5 ± 3.6 vs. 1.0 ± 3.6 mm), FH restoration at L4–5 (3.0 ± 3.5 vs. −0.1 ± 4.4 mm), SL (4.1 ± 6.4 vs. −2.1 ± 8.1°), LL (4.1 ± 7.0 vs. −2.3 ± 12.6°) and PI–LL mismatch (4.1 ± 7.0 vs. 2.3 ± 12.6°). Fusion, migration, subsidence, and ASD incidence were not significantly different; however, neurological deficits were more common in LLIF (9 vs. 3).

Nakashima et al. [46] assessed 2-year SL angle, DH, LL, PT, C7 SVA, and TK of PLIF and LLIF. In two-level fusions, LLIF produced greater changes in SL angle (4.8 ± 4.0 vs. 2.6 ± 3.2°), DH (4.0 ± 1.5 vs. 2.4 ± 1.9 mm), LL (8.4 ± 7.0 vs. 2.1 ± 6.7°), and PI–LL (−9.0 ± 7.3 vs. −3.4 ± 7.4). In three-level fusions, LLIF had greater improvement in LL (12.1 ± 11.1 vs. 4.2 ± 9.1°), PI–LL (−11.2 ± 11.3 vs. −3.0 ± 9.3), PT (−6.4 ± 4.9 vs. −2.5 ± 5.3°), and TK (7.8 ± 11.8 vs. −0.3 ± 9.7°).

Yoon et al. [52] assessed radiographic parameters of OLIF and TLIF. The authors found that the OLIF group had greater postoperative cage height (14.3 ± 1.5 vs. 11.5 ± 1.2 mm), cage angle (16.0 ± 5.6 vs. 5.6 ± 2.1), change in anterior DH (6.9 ± 3.2 vs. 4.7 ± 2.9 mm), correction of SL (−13.8 ± 7.5 vs. −7.4 ± 9.1°), and change in disk angle (−9.2 ± 5.2 vs. −5.1 ± 5.1°). The TLIF group had greater changes in posterior DH (2.4 ± 2.6 vs. 1.0 ± 2.4 mm) and FH (1.1 ± 2.8 vs. 0.2 ± 2.9 mm). There were no differences in PI, LL, PI–LL mismatch, PT, SVA correction, or complication rates.

#### 3.4.2. Procedural Techniques

Song et al. [49] assessed PLIF with a cage vs. autogenous bone alone. The authors found no significant differences in leg pain, back pain, ODI, DH, LL correction, fusion rate, instrumentation failure, or subsidence between the two fusion techniques. Li et al. [43] matched TLIF patients with a n-HA/PA66 cage to PEEK cages. The authors reported that the n-HA/PA66 cage had a lower incidence of ASD (14.58% vs. 33.33%); however there were no differences in fusion rate, intervertebral space height, segmental angle, LL, VAS, ODI, or complication rates. Zhao et al. [54] assessed a unilateral vs. bilateral incision for open and percutaneous PSF in the TLIF procedure. Despite increased operative time (163.07 ± 37.94 vs. 147.15 ± 37.96, minutes), estimated blood loss (235.61 ± 88.77 vs. 190.57 ± 73.04, mL), and radiation exposure time (24.13 ± 1.74 vs. 17.71 ± 1.99 s) in the unilateral incision cohort, there were no differences in length of stay or clinical outcomes.

#### 3.4.3. MIS vs. Open TLIF

Gu et al. [33] (248.4 ± 94.3 vs. 576.3 ± 176.2), Lee et al. [42] (527.41 ± 219.66 vs. 865.81 ± 525.09), and Zhang et al. [53] (254.1 ± 23.97 vs. 450.7 ± 36.23) reported that MIS TLIF had less intraoperative blood loss (mL). Gu et al. [33] (9.3 ± 3.7 vs. 12.1 ± 3.6) and Zhang et al. [53] (6 ± 0.92 vs. 8 ± 2.2) reported a shorter length of stay (days) in the MIS cohort, but Lee et al. [42] found no difference. Lee et al. [42] (167.33 ± 37.54 vs. 216.58 ± 40.41) and Zhang et al. [53] (136.3 ± 27 vs. 148.4 ± 32.99) reported shorter operative time (minutes) in MIS, but Gu et al. [33] reported no difference. All authors found no difference in improvement of back pain, leg pain, ODI, fusion, improvement of sagittal parameters, pelvic parameters, or PROMs. Zhang et al. [53] reported that MIS TLIF had a lower complication rate (0% vs. 24.32%).

#### 3.4.4. Unilateral vs. Bilateral Pedicle Screw Fixation Within TLIF

Gu et al. [34] (154.6 ± 22.1 vs. 185.9 ± 27.2), Liu et al. [44] (126.8 ± 25.9 vs. 198.1 ± 36.0), and Zhang et al. [32] (208 ± 36.51 vs. 257 ± 34.79) reported a shorter operative duration (minutes) when using unilateral fixation. Similarly, Gu et al. [34] (190.9 ± 61.0 vs. 256.2 ± 96.8), Liu et al. [44] (247.5 ± 96.4 vs. 345.6 ± 154.9), and Zhang et al. [32] (391 ± 134.75 vs. 546 ± 161.150) reported less blood loss (mL) when using unilateral fixation. Liu et al. [44] and Zhang et al. [32] reported no differences in radiographic or clinical outcomes, whereas Gu et al. [34] found greater improvements in cobb angle of the whole lumbar (2.05 ± 1.15 vs. 1.96 ± 0.81) and lower improvement in whole lumbar lordosis (7.26 ± 2.37 vs. 8.46 ± 2.74) through unilateral fixation.

### 3.5. Risk of Bias

**Figure 2:**
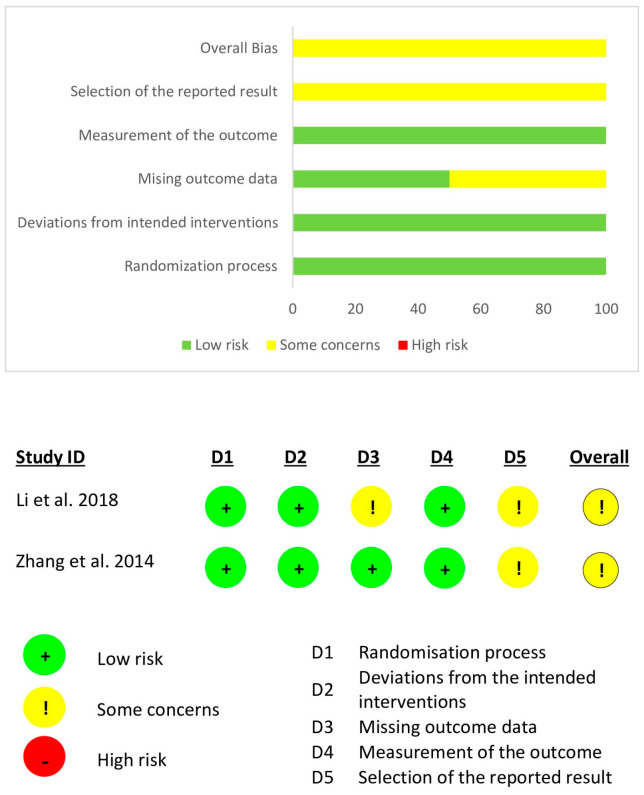
Risk of bias assessment of randomized studies [31,32].

All non-randomized studies underwent appraisal using the MINORS criteria (Table 1). Five studies were rated as low-quality evidence [57,59,69,72,75], 25 studies were rated as fair-quality evidence [37,38,39,40,45,48,50,51,52,55,56,58,60,61,62,63,64,65,66,67,68,70,71,73,74], and 13 studies were rated as good-quality evidence [33,34,35,36,40,42,43,44,46,47,49,53,54]. All individual MINORS scores can be found in Table 1. Two randomized studies assessed by the Cochrane RoB-2 tool indicated some concerns of bias (Figure 2).

## 4. Discussion

### 4.1. Summary of Analysis

The findings of this systematic review provide a comprehensive resource of operative details, as well as radiographic and clinical outcomes within lateral and posterior multilevel LIF techniques, namely the LLIF, OLIF, TLIF, and PLIF procedures. Quantitative procedural comparison of clinical outcomes was not conducted due to significant heterogeneity; therefore, the reported data was aggregated to qualitatively present the current multilevel LIF literature.

### 4.2. Operative Details

Surgical details were found to be highly heterogeneous. While posterior instrumentation was common, there was significant variation in the placement and decision for unilateral or bilateral fixation, similarly applying to the use of grafts and BMP (Table S2). LLIF was found to have the greatest mean operative duration of 272.8 min, with the lowest being TLIF at 198.7 min; however, limited LLIF data undermines the accuracy of this finding. For example, LLIF operative time may be inflated, as this measurement came from a single study utilizing a lateral-to-prone repositioning for PSF [36]. PLIF had the greatest intraoperative blood loss of 584.67 mL, whereas LLIF had the lowest intraoperative blood loss of 229 mL. While still subject to bias as a result of the low LLIF patient population, this finding aligns with LLIF’s percutaneous approach and minimal extradiscal dissection [76]. However, a lack of control and inherent variability of factors contributing to blood loss must be considered [77]. The reported LOS was found to cluster at extreme values. TLIF (5.09 days) was similar to LLIF (5.5 days), whereas OLIF (12.9 days) and PLIF (14.25 days) had a much longer LOS. While OLIF has limited evidence, the data on the predominantly open PLIF group correlates with recent evidence of increased LOS in non-MIS techniques [78]. The collected operative duration, blood loss, and LOS values resemble data trends from a recently published meta-analysis on LLIF vs. alternatives for treating degenerative spinal conditions [24].

### 4.3. Patient-Reported Outcome Measures

The collection of PROMs varied significantly, with no apparent standardization. The ODI and VAS pain scales were the greatest reported by a considerable margin; however, all collected PROMs were included for visualization (Table S5). The ODI scores were only reported for TLIF, PLIF, and LLIF, with TLIF having the greatest ODI score gain of 33.68 and LLIF having the lowest score gain of 21.04. Postoperative VAS leg pain score gain was the greatest for TLIF at 5.39, whereas LLIF had the lowest score gain, 1.62. The reported VAS back pain followed a similar trend, with TLIF having the greatest score gain, 4.67, and LLIF having the lowest score gain, 3.58. Interestingly, all score gain differences between LLIF and TLIF either approached or surpassed the MCID of 12.8, 1.6, and 1.2 for ODI, leg pain, and back pain, respectively [79]. However, a lack of statistical analysis precludes conclusions on this difference. These values differ from a meta-analysis finding that LLIF had favorable postoperative ODI scores resistant to change when using meta-regression to assess the influence of operated level [24].

### 4.4. Radiographic Outcomes

Radiographic outcomes can be further segmented into fusion rates and alignment results. Fusion rates were the greatest for OLIF, 96.7%, and the lowest for TLIF, 92.95%. However, the OLIF results came from one study. The data within this review found that the sagittal alignment parameters showed improvement for all fusion approaches, including lumbar lordosis, pelvic incidence, and SVA (Table 4). While there is currently some literature questioning the correlation of solid arthrodesis with desirable outcomes, the ample data within this review and the PRO score gains surpassing the MCID appeared to support this correlation [80,81,82].

### 4.5. Complications

As a result of small cohort size, LLIF had a skewed complication rate much higher than other procedures. The complication rates of the OLIF procedure closely resembled those of TLIF and PLIF, for which standard deviations overlapped substantially. As expected, dural tears were more common for PLIF (4.04%) than TLIF (2.91%), and psoas weakness occurred at the greatest rate with LLIF (42.86%). Neurologic complications, defined as weakness or numbness, were much more common in LLIF (33%) and to a lesser extent in PLIF (9.8%). These events were all described as transient episodes with no permanent sequelae, with the exception of one LLIF patient with motor deficits (L2 deficit, MRC grade 4) at the 2-year follow up [36]. Previous research has presented conflicting results. An et al. conducted a meta-analysis on OLIF and PLIF, finding that complications rates were significantly lower in the OLIF than in the PLIF group [23]. In a meta-analysis comparing OLIF and MIS TLIF, Zhang et al. found no differences [25]. When LLIF was compared against posterior alternatives, there was a significant risk reduction in the lateral procedure, which was resistant to meta-regression [24]. Currently, the most high-evidence analyses comparing lateral and posterior LIF procedures contain primarily single-level data with a combination of procedures, which can introduce confounding effects and limit our ability to contextualize multilevel LIF results.

### 4.6. Procedural Comparison

Within comparative evidence assessing LIF procedures, all three produced similar conclusions of optimal clinical performance through a lateral approach. When comparing MIS LLIF to MIS TLIF, Chong et al. [36] noted lower blood loss, DH, and FH restoration at the cost of increased neurological deficits in the LLIF procedure. The authors state that these results are somewhat expected, as LLIF facilitates disk distraction at the midline, with cages spanning the epiphyseal ring [83]. Notably, LLIF was performed on L3–L5, which could be the cause of the observed favorable LL restoration, although favorable sagittal parameters did not result in an improvement of clinical outcomes.

Assessing 2-year radiographic outcomes, Nakashima et al. [46] found that, in addition to DH, the LLIF procedure outperformed PLIF in both two- and three-level fusions for many radiographic parameters. Similar to Chong et al. [36], the authors agreed that this may be as a result of advantages inherent to LLIF such as access to disk space and endplate preparation. The authors added that LLIF interbody cages are unique in that they provide an extension–distraction moment, permitting the enlargement of the interbody space and DH; however, a 2-year follow up may be too short to see a manifestation of this advantage [84].

Yoon et al. [52] analyzed the OLIF procedure against TLIF; the authors found these results were largely limited to DH, angle, and SL, with no difference in sagittal alignment or complication rates. Despite affirming previously documented findings of increased cage and DH through a lateral approach such as the OLIF procedure, the authors noted no differences in sagittal alignment, attributing this finding to the use of bilateral facetectomies within TLIF, which achieved posterior column shortening and lordosis by rod assembly with compressive force.

### 4.7. Procedural Techniques

Three studies assessed differences in techniques within multilevel LIF. In PLIF, for two-level isthmic-spondylolisthesis, Song et al. [49] assessed the advantages of a single PEEK cage against the use of autogenous bone in procedures using posterior instrumentation. The authors concluded that despite prior literature indicating the necessity of an interbody cage to restore disk height and supply structural support [85], the use of autogenous bone obtained during decompression performed equally. Li et al. [43] conducted a 6-year minimum follow-up study of n-HA/PA66 vs. PEEK cages for multilevel degenerative disease. The cohorts were matched for demographics, including body mass index (BMI), Charlson comorbidity index (CCI), bone mineral density (via T-score), and excluded patients with preoperative Pfirrmann grades above 3. The authors reported that outcomes remained homogenous; however, the incidence of ASD was twice as high in the PEEK cage cohort, likely as a result of n-HA/PA66’s advantages at the material–tissue interface. Lastly, Zhao et al. [54] assessed the impact of unilateral long or bilateral short decompression incisions when using open and percutaneous pedicle screw fixation within TLIF. The authors reported that a unilateral long incision resulted in a longer operative duration and blood loss with no advantages in LOS or clinical outcomes.

### 4.8. MIS vs. Open

We examined three studies that compared MIS vs. open TLIF procedure [33,42,53]. All three studies reported a reduction in intraoperative blood loss as a result of MIS, whereas only two studies, respectively, found a difference in length of stay [33,53] and operative time [42,53], favoring MIS TLIF. In all three studies, no operative details favoring MIS translated into an improvement of PROM scores, fusion rates, or radiographic parameters. Zhang et al. [53] found a lower complication rate in the MIS TLIF cohort, stemming from less retraction. This finding may also be attributable to the increased follow-up length of 5 years, in comparison to the 2-year follow up and 1-year follow up in Gu et al. [33] and Lee et al. [42], respectively. When investigating study methodology, all MIS procedures used a single interbody cage compared to two cages in Open TLIF, a disadvantage of MIS TLIF that may affect fusion over a longer follow-up [86,87]. Fusion was homogenously determined by Bridwell’s scale [88], and despite varying this interbody cage difference, both MIS and open groups demonstrated a high fusion rate with a minimal non-significant difference.

### 4.9. Unilateral vs. Bilateral Pedicle Screw Fixation

We examined three studies comparing unilateral vs. bilateral PSF within the TLIF procedure [32,34,44]. These studies reported a reduction in operative time and blood loss when using unilateral PSF. This finding seems intuitive, as placing more screws and a second rod would require greater operative time and result in increased blood loss. Gu et al. [34] found that the unilateral fixation cohort produced favorable improvements in the whole lumbar cobb angle and unfavorable results in lumbar lordosis; however, the differences in clinical outcomes were strongly non-significant. While fusion rates were non-significant, the assessment methods were found to be somewhat heterogenous: Gu et al. [34] assessed with plain X-rays at 12 months, Liu et al. [44] assessed with assistance of flexion-extension X-rays and CT scans, and Zhang et al. [32] assessed with annual CT scans. While previous literature on LIF has found that flexion–extension X-rays can be a valuable tool in identifying lumbar instability, a lack of standardization and inconsistent use of CT scans limit robust evidence for true differences in fusion rate [89,90].

### 4.10. Bias Within the Literature

The review process revealed a concerning aspect within the multilevel LIF literature. A vast majority of the literature focused on posterior approaches, with 42 studies, while LLIF and OLIF were only discussed in six. This may be due to the technical nature of LLIF and OLIF in addressing complex multilevel pathology; however, LLIF publications are increasing at an accelerating rate [91]. Additionally, 21 of the included studies were level 4 evidence. Individual study bias analysis with the MINORS tool found that most included studies were low- or fair-quality evidence, whereas a minority were classified as good-quality. Similarly, some concerns of bias were found in both included RCTs. Beyond an apparent publication bias favoring the posterior LIF approaches, the assessment of study quality further highlights the deficiencies within the available evidence. While a few outcomes indicated favorable results through lateral approaches, there is a lack of evidence to definitively claim a broadly superior procedure between LIF alternatives. Furthermore, as operative level is often pooled, the literature limits subgroup analysis to determine optimal clinical benefit within a specified lumbar segment.

### 4.11. Cost

If heterogeneity of outcomes continues to limit the ability to determine a broadly favorable LIF procedure, attention can be shifted towards cost reduction. At a 2-year time horizon, TLIF had a favorable cost–utility metric (lower cost/QALY) when considering direct costs of the procedure; however, LLIF became more favorable when indirect costs such as productivity loss were included [92]. A subgroup analysis was conducted on MIS vs. open procedures finding that at a time horizon of 1 year, MIS was favorable, but when the time horizon was extended to 2 years, the costs approached similarity [93,94]. This suggests that there is an immediate quality-of-life benefit as a result of reduced blood loss or LOS, findings that were applicable to the multilevel LIF literature.

### 4.12. Limitations and Future Research

This study is not without limitations. Although this study utilized strict exclusion criteria, multilevel LIF literature is inherently heterogenous. This limited our ability to quantitatively compare the LLIF, OLIF, TLIF, and PLIF procedures. Furthermore, inconsistent reporting and a publication bias within the literature increases the difficulty of reporting accurate values on LLIF and OLIF. This is especially apparent within the OLIF literature, where reported procedural characteristics and outcome values come from a very limited dataset. Limited reporting of consistent clinical outcomes is a substantial limitation of the multilevel LIF literature and present study, substantially weakening conclusions regarding the clinical effectiveness of the procedures. This was notable for PROMs, where almost the entirety of the evidence was within the TLIF and PLIF cohorts. Clinical heterogeneity within surgical techniques, assessment of radiographic outcomes, and level 4 evidence introduced bias into the reported pooled outcomes. Heterogeneity of included studies limited the ability for further subgroup analysis by study quality beyond the stratification completed within the present study. Additionally, there was significant variation in clinical indications across the included studies and further stratification by indication was not methodologically feasible. This significantly limits the ability to draw meaningful conclusions by surgical approach or indication. It is important to note that a lack of intersection between level 3 or higher evidence across the collected outcomes necessitated the inclusive nature of the pooled outcomes. As higher-level of evidence studies did not consistently report on the same variables, a reliance on level 4 evidence was required to provide a comprehensive overview of the current clinical landscape.

Future studies should prioritize quantitative procedural comparison on a subset of high-level evidence to ensure that conclusions can remain robust by employing study designs such as randomized controlled trials or multicenter prospective cohorts. These studies should leverage the comprehensive data gaps identified through our exhaustive search and grouping of the available literature as a roadmap to target specific approaches and indications, thereby providing the high-level evidence necessary to impact clinical decision-making. Similarly, future studies can expand their investigation to the anterior procedure (ALIF) or segment analysis by operated levels. Future reviews can prioritize high-quality evidence of lateral procedures to reduce bias or confounding effects. As future studies look to iterate on the limitations of the present data and study, emergent technology within lumbar interbody fusion can be considered in conjunction. Recent literature has investigated the use of robotic assistance within lumbar procedures, finding that robot guidance can outperform freehand and CT-guided placement of pedicle screws [95,96], with evidence indicating a reduction in surgical revision due to screw mispositioning [95]. Both meta-analyses call for high-level evidence to further confirm the benefits of robotic assistance.

## 5. Conclusions

While this comprehensive review identified significant heterogeneity, it is the first to consolidate all reported outcomes in the current multilevel LIF literature. Despite pooled outcome measures favoring the TLIF procedure, a publication bias towards posterior LIF approaches was observed. Conversely, available comparative evidence contradicted these pooled findings, reporting advantages for the LLIF and OLIF procedures over their posterior counterparts. Given the inconsistent evidence, results should be interpreted with caution. It is essential that future research utilizes robust study design to provide data and render insight to aid clinical decision-making of lumbar interbody fusion for multilevel pathology.

## Figures and Tables

**Table 1:** Study characteristics.

Study	Study Design	MINORS Score	LIF Procedure	Mean Follow Up ± SD (Range), Months	Mean Age ± SD (Range), y	Patient N	Male N	Fused Levels N	Operated Levels
Ahmadian 2015 [55]	Case series	8.5	LLIF	14.57 ± 4.86 (12–24)	63.76 ± 10.35 (33–77)	21	7	2.71 (2–4)	L2–L5 8 L1–L4 3 L1–L5 2 L2–L4 3 L3–L5 5
Aono 2018 [56]	Case series	11	PLIF	57.6 (24–132)	73.1 ± 7.8 (45–85)	48	10	2	L2–4 5 L3–5 33 L4–S1 10
Chong 2024 [36]	Retrospective cohort	19.5	LLIF TLIF	24	LLIF: 68.4 ± 6.8 TLIF: 65.3 ± 9.3	LLIF: 24 TLIF: 29	LLIF: 9 TLIF: 9	2	LLIF: L3–L5 24 TLIF: L3–L5 29
Claus 2021 [37]	Retrospective cohort	11.5	TLIF	24	64.87 ± 8.3	3120 total 3117 complication analysis 94 for PRO analysis	1499	2.46 (2–4)	NR
Couture 2004 [57]	Case series	7	PLIF	26.1 (17–32)	NR	12	NR	2.58 (2–4)	NR
Du 2019 [38]	Retrospective cohort	15.5	TLIF	34.2 ± 11.8 (24–73)	54.4 ± 9.4	38	15	2	L4–S1 38
Ferraro 2023 [58]	Case series	8.5	TLIF	30 (24–58)	52.3 ± 12.2	14	8	2	L4–S1 14
Fujimori 2020 [39]	Retrospective cohort	15	PLIF (lumbar) PLIF (lumbosacral)	Lumbar: 25.45 ± 3.72 Lumbosacral: 24.95 ± 4.27	Lumbar: 68 ± 7 Lumbosacral: 71 ± 8	Lumbar: 48 Lumbosacral: 25	Lumbar: 9 Lumbosacral: 10	2	Lumbar: L3–L5 48 Lumbosacral: L4–S1 25
Gu 2014 [33]	Prospective cohort	16.5	TLIF (MIS) TLIF (open)	MIS: 20.6 ± 4.5 Open: 20.0 ± 3.3	MIS: 66.4 ± 6.7 Open: 64.1 ± 7.8	MIS: 44 Open: 38	MIS: 19 Open: 15	2	MIS: L3–L5 13, L4–S1 31 Open: L3–L5 14, L4–S1 24
Gu 2015 [34]	Prospective cohort	17.5	TLIF (unilateral) TLIF (bilateral)	Unilateral: 32.1 ± 7.5 Bilateral: 31.7 ± 8.0	Unilateral: 64.5 ± 8.0 Bilateral: 66.1 ± 7.1	Unilateral: 35 Bilateral: 39	Unilateral: 17 Bilateral: 21	2	Unilateral: L3–L5 15, L4–S1 20 Bilateral: L3–L5 16 L4–S1 23
Guppy 2021 [40]	Retrospective cohort	18	PLIF	63.13 ± 38.64	NR	440	NR	2.05 (2–3)	L3–L5: 53 L4–S1: 373 L3–S1: 14
Hackenberg 2005 [41]	Retrospective cohort	11	TLIF	46 (36–64)	NR	13	NR	2.15 (2–3)	L3–S1
Hioki 2005 [59]	Case series	7	PLIF	43.2 ± 20.4 (24–89)	59.7 ± 7.9 (32–73)	19	8	2	L3–L5: 15 L4–S1: 4
Kalinin 2021 [35]	Prospective cohort	19	TLIF (ARP) TLIF (traditional)	ARP: 17.33 ± 7.8 Traditional: 19 ± 8.58	ARP: 57 ± 19.7 Traditional: 54.67 ± 21.05	ARP: 24 Traditional: 29	ARP: 14 Traditional: 18	2	ARP: L2–L4:1 L3–L5: 6 L4–S1: 17 Traditional: L2–L4: 2 L3–L5: 7 L4–S1: 20
Kim 2011 [60]	Case series	9	PLIF	25.3 ± 7.161 (12–43)	59.1 ± 12.71 (23–78)	42	14	2.24 (2–3)	NR
Kurra 2018 [61]	Case series	10	TLIF	60	NR	12		2.09 (2–3)	NR
Lee 2016 [42]	Retrospective cohort	18	TLIF (MIS) TLIF (open)	12	MIS: 60.55 ± 13.61 Open: 65.06 ± 6.81	MIS: 27 Open: 43	MIS: 8 Open: 16	MIS: 2.11 (2–3) Open: 2.29 (2–3)	NR
Li 2024 [43]	Retrospective cohort	18	TLIF (n-HA/PA66) TLIF (PEEK)	n-HA/PA66: 84.81 ± 9.60 PEEK: 87.48 ± 9.40	n-HA/PA66: 56.02 ± 5.13 PEEK: 56.92 ± 6.11	n-HA/PA66: 48 PEEK: 48	n-HA/PA66: 18 PEEK: 21	n-HA/PA66: 2.61 (2–4) PEEK: 2.52 (2–4)	L1–L5
Li 2018 [31]	RCT	NA	PLIF	48	51.8 ± 6.8	46	24	3.11 (3–4)	NR
Liu 2016 [44]	Retrospective cohort	17	TLIF (UPS) TLIF (UPSFS) TLIF (BPS)	UPS: 46.4 ± 6.0 UPSFS: 45.6 ± 8.8 BPS: 46.4 ± 5.5	UPS: 59.16 ± 9.67 UPSFS: 59.32 ± 12.80 BPS: 58.91 ± 8.51	UPS: 22 UPSFS: 28 BPS: 34	UPS: 8 UPSFS: 12 BPS: 12	2	UPS: L2–L4 1, L3–L5 11, L4–S1 10 UPSFS: L2–L4 0, L3–L5 14, L4–S1 14 BPS: L2–L4 1, L3–L5 18, L4–S1 15
Lu 2015 [45]	Retrospective cohort	13	PLIF (Group A, solo) PLIF (Group B, DIAM)	Group A: 41.5 ± 8.6 (24–48) Group B: 41.2 ± 7.2 (24–48)	Group A: 59.1 ± 8.6 (35–81) Group B: 64.5 ± 7.2 (46–78)	Group A: 42 Group B: 49	Group A: 14 Group B: 16	2.59 (2–4)	Group A: L4–S1 21, L3–S1 17, L2–S1 4 Group B: L4–S1 10, L3–S1 30, L2–S1 9
Luan 2022 [62]	Case series	8	TLIF	>12	65.45 ± 8.85	36	10	2	L3–L5 21 L4–S1 15
Mao 2014 [63]	Case series	9.5	TLIF	36	59.6 ± 4.85 (48–72)	98	61	2	NR
Min 2013 [64]	Case series	9.5	TLIF	22.86 ± 7.55 (18–52)	60.28 ± 9.99 (32–77)	58	18	2.31 (2–3)	NR
Nakashima 2019 [46]	Retrospective cohort	16	PLIF LLIF	24	PLIF: 68.2 ± 11.09 LLIF: 72.99 ± 4.81	PLIF: 35 LLIF: 43	PLIF: 17 LLIF: 18	PLIF: 2.4 (2–3) LLIF: 2.35 (2–3)	PLIF: L2/3 14, L3/4 35, L4/5 35 LLIF: L2/3 16, L3/4 43, L4/5 42
Nourian 2019 [65]	Case series	8.5	LLIF	20	65	22	NR	2	L3–L5
Okuda 2018 [66]	Case series	9.5	PLIF	72	NR	55	NR	2	NR
Ormond 2016 [67]	Retrospective cohort	8	TLIF	33.28 ± 16.1	45.57 ± 8.56	7	4	(2–3)	L3–L5, L4–S1, L3–S1
Park 2023 [47]	Retrospective cohort	16.5	PLIF	12	NR	38	NR	2.1 (2–3)	L2/3–L5/S1
Rouben 2011 [68]	Case series	11.5	TLIF	>36 (36–60)	NR	45	NR	2	L2–L4 3 L3–L5 7 L4–S1 35
Sakaura 2018 [48]	Retrospective cohort	14	PLIF	35.4 ± 11.4	68.3 ± 9.6	20	6	2	L2–L4, L3–L5, L4–S1
Salehi 2004 [69]	Case series	5	TLIF	15.5 ± 10.71	38.91 ± 13.08	11	7	2	L4–S1 11
Song 2015 [49]	Retrospective cohort	16.5	PLIF (autogenous bone chips, Group 1) PLIF (cage, Group 2)	Group 1: 27.2 Group 2: 26.8	Group 1: 48.8 Group 2: 50.4	Group 1: 29 Group 2: 25	Group 1: 8 Group 2: 7	2	Group 1: L4–S1 29 Group 2: L4–S1 25
Song 2017 [70]	Case series	9	PLIF	33.6 ± 11.76 (24–72)	51.3 ± 10.25 (36–67)	32	9	2	L3–L5: 2 L4–S1: 30
Takahashi 2019 [71]	Case series	13.5	PLIF	67.2 ± 26.46 (24–132)	72 ± 7.35 (55–85)	33	9	2	L3–5: 33
Talia 2015 [72]	Case series	6	TLIF	12	65.5 ± 7.48	12	2	2.08 (2–3)	L3–L5, L4–S1
Tsai 2021 [73]	Case series	10.5	TLIF	24	70.08 ± 8.47	12	0	2	L3–L5: 12
Xi 2020 [50]	Retrospective cohort	13.5	OLIF (nonobese) OLIF (obese)	Nonobese: 14.15 ± 9.79 Obese: 18.88 ± 9.97	Nonobese: 65.15 ± 11.81 Obese: 60.38 ± 13.02	Nonobese: 53 Obese: 24	Nonobese: 14 Obese: 11	Nonobese: 2.89 ± 0.954 Obese: 2.88 ± 0.797	Nonobese: L4–S1 26, L3–S1 8, L2–S1 18, L1–S1 1 Obese: L4–S1 9, L3–S1 9, L2–S1 6
Yang 2014 [51]	Retrospective cohort	14.5	PLIF	26.04 ± 9.12	54.69 ± 3.26	45	21	(2–3)	L3–L4: 2 L4–L5:20 L5–S1: 17 L3–L5: 2 L4–S1: 4
Yoo 2014 [74]	Case series	8.5	TLIF	25.92	60.53 ± 10.46	45	24	2.42 (2–5)	L3–L5: 2 L4–S1: 27 L2–L5: 3 L3–S1: 11 L2–S1: 1 L1–S1: 1
Yoon 2023 [52]	Retrospective cohort	14.5	OLIF TLIF	OLIF: 28.8 ± 15.6 TLIF: 51.6 ± 33.6	OLIF: 66.0 ± 8.4 TLIF: 66.3 ± 9.6	OLIF: 60 TLIF: 58	OLIF: 23 TLIF: 19	OLIF: 2.33 (2–3) TLIF: 2.12 (2–3)	OLIF: L2/L3 16, L3/4 57, L4/5 59, L5/S1 8 TLIF: L1/L2 1, L2/L3 6, L3/4 31, L4/5 55, L5/S1 30
Zhang 2014 [32]	RCT	NA	TLIF (unilateral) TLIF (bilateral)	Unilateral: 25.6 ± 4.41 (18–36) Bilateral: 25.6 ± 4.41 (18–36)	Unilateral: 59.4 ± 10.2 Bilateral: 55.7 ± 11.6	Unilateral: 33 Bilateral: 35	Unilateral: 14 Bilateral: 25	2	Unilateral: L4–S1 20, L3–L5 12, L2–L4 1 Bilateral: L4–S1 23, L3–L5 10, L2–L4 2
Zhang 2018 [75]	Case series	7	PLIF	50.04 ± 15 (24–78.96)	61 ± 8.76 (44–77)	24	NR	2	L2–L4: 1 L3–L5: 11 L4–S1: 12
Zhang 2022 [53]	Retrospective cohort	17.5	TLIF (MIS) TLIF (open)	MIS: 72.2 ± 3.2 Open: 76.5 ± 4.2	MIS: 59.9 ± 6.9 Open: 61.8 ± 5.6	MIS: 45 Open: 37	MIS: 25 Open: 21	MIS: 2.24 (2–3) Open: 2.32 (2–3)	NR
Zhao 2018 [54]	Retrospective cohort	17.5	TLIF (unilateral incision) TLIF (bilateral incision)	Unilateral: 21.87 ± 8.90 Bilateral: 24.56 ± 10.08	Unilateral: 61.24 ± 14.16 Bilateral: 62.13 ± 15.05	Unilateral: 62 Bilateral: 67	Unilateral: 30 Bilateral: 35	2	Unilateral: L3–L5 26, L4–S1 36 Bilateral: L3–L5 28, L4–S1 39

RCT, randomized controlled trial, MINORS, methodological index for nonrandomized studies; LLIF, lateral lumbar interbody fusion; OLIF, oblique lateral interbody fusion; PLIF, posterior lumbar interbody fusion; TLIF, transforaminal lumbar interbody fusion; SD, standard deviation; MIS, minimally invasive; UPS, unilateral pedicle screw; UPSFS, unilateral pedicle screw and facet screw; BPS, bilateral pedicle screw; ARP, advanced recovery protocol; DIAM, device for intervertebral assisted motion; n-HA/PA66, nanohydroxyapatite/polyamide-66; PEEK, polyether ether ketone; NR, not reported; y, years.

**Table 2:** Summary of weighted pooled operative details.

	TLIF			PLIF			LLIF			OLIF		
Operative Detail	Study N	Patient N	Mean ± SD (Range)	Study N	Patient N	Mean ± SD (Range)	Study N	Patient N	Mean ± SD (Range)	Study N	Patient N	Mean ± SD (Range)
Operative Duration, min	13	3948	198.7 ± 77.83 (141.76–308.8)	7	305	226.65 ± 46.63 (176.98–350.4)	1	24	272.8 ± 82.4 ^a^	2	137	233.77 ± 66.11 (226.8–239.2)
Intraoperative Blood Loss, mL	13	3981	402.83 ± 305.46 (131.51–802.2)	8	347	584.67 ± 255.68 (327.4–1277.6)	1	24	229 ± 125.6	2	137	375.92 ± 171.92 (96.11–735)
Length of Stay, Days	12	3899	5.09 ± 2.5 (3.75–16.8)	3	173	14.25 ± 3.02 (8.5–19.34)	1	24	5.5 ± 2.8	1	60	12.9 ± 4.3

^a^ Operative duration for LLIF refers to a lateral-to-prone procedure for posterior instrumentation; mL, milliliters.

**Table 3:** Summary of weighted pooled patient reported outcome measures.

	TLIF			PLIF			LLIF		
PROM	Study N	Patient N	Mean ± SD (Range)	Study N	Patient N	Mean ± SD (Range)	Study N	Patient N	Mean ± SD (Range)
ODI	16	971	33.68 ± 6.43 (15.1–55.47)	3	141	30.58 ± 5.01 (10.8–43.32)	2	45	21.04 ± 7.05 (15.98–25.4)
VAS Leg Pain	13	839	5.39 ± 0.66 (3.6–6.7)	4	214	4.19 ± 0.97 (3.7–5.6)	1	24	3.41 ± 0.07
VAS Back Pain	18	1077	4.67 ± 0.79 (2.4–6.6)	7	312	4.54 ± 0.78 (3.29–6.63)	2	45	3.58 ± 0.67 (2.86–4.22)

ODI, Oswestry disability index; VAS, visual analog scale; SD, standard deviation.

**Table 4:** Summary of weighted pooled radiographic outcomes.

	TLIF			PLIF			LLIF			OLIF		
Outcome	Study N	Patient N	Mean ± SD (Range)	Study N	Patient N	Mean ± SD (Range)	Study N	Patient N	Mean ± SD (Range)	Study N	Patient N	Mean ± SD (Range)
Fusion Rate, %	15	958	92.95 ± 5.28 (81.82–100)	13	881	94.37 ± 6.78 (83.3–100)	3	67	94.02 ± 7.19 (86–100)	1	60	96.7
Thoracic Kyphosis, °	3	144	3.56 ± 3.78 (1.18–8.2)	2	68	1.01 ± 0.98	1	43	3.39	0	0	NR
Lumbar Lordosis, °	12	737	6.59 ± 3.63 (2.3–15.8)	8	309	5.49 ± 2.76 (1.7–10.4)	2	67	7.6 ± 3.67 (4.5–9.69)	1	60	8.1 ± 2.4
Segmental Lordosis, °	6	423	2.33 ± 1.64 (0.1–5.06)	1	33	0.6 ± 1.3	1	24	4.3 ± 1.2	0	0	NR
Pelvic Incidence, °	5	254	0.71 ± 0.55 (0.13–1.6)	3	111	1.79 ± 1.4 (0.6–3.2)	0	0	NR	0	0	NR
SVA, °	4	164	29.86 ± 16.88 (16.04–49.76)	2	68	25.53 ± 17.55 (13.5–38.3)	1	43	21.11	1	60	30.4 ± 32.2
Pelvic Tilt, °	7	341	3.51 ± 3.29 (0.3–10.5)	2	68	1.7 ± 1.65 (0.5–2.83)	2	67	3.95 ± 0.41	1	60	1.3 ± 0.3
Sacral Slope, °	5	254	4.67 ± 2.55 (2.89–9.4)	2	79	3.08 ± 2.4 (1.1–4.5)	0	0	NR	0	0	NR
PI–LL Mismatch, °	4	219	3.98 ± 1.79 (2.3–6.4)	2	68	2.83 ± 0.59 (2.4–3.24)	2	67	8.01 ± 3.09 (5.4–9.7)	1	60	8 ± 3.6

NR, not reported.

## Data Availability

No new data were created or analyzed in this study. Data sharing does not apply to this article.

## Supplementary Materials

The following supporting information can be downloaded at: https://www.mdpi.com/article/10.3390/jcm15062289/s1, Table S1: Full Literature Search Strategy; Table S2: Individual study indications, surgical details, author conclusions, and limitations; Table S3: Pooled Complication Events and Weighted Rates; Table S4: Complications Reported as Other; Table S5: Collected Patient Reported Outcome Measures; PRISMA 2020 Checklist.

## Abbreviations

The following abbreviations are used in this manuscript:

LIF	lumbar interbody fusion
MIS	minimally invasive
PSF	pedicle screw fixation
PLF	posterolateral fusion
LLIF	lateral lumbar interbody fusion
XLIF	extreme lateral interbody fusion
DLIF	direct lateral interbody fusion
PLIF	posterior lumbar interbody fusion
TLIF	Transforaminal lumbar interbody fusion
OLIF	oblique lateral interbody fusion
PRISMA	Preferred Reporting Items for Systematic Reviews and Meta-Analyses
CENTRAL	Cochrane Central Register of Controlled Trials
PROM	patient-reported outcome measures
ASD	adjacent segment disease
MINORS	methodological index for non-randomized studies
RoB-2	Cochrane risk-of-bias tool
RCT	randomized controlled trial
UPS	unilateral pedicle screw
UPSFS	unilateral pedicle screw and facet screw
BPS	bilateral pedicle screw
ARP	advanced recovery protocol
DIAM	device for intervertebral assisted motion
n-HA/PA66	nanohydroxyapatite/polyamide-66
PEEK	polyether etherketone
BMP	Bone morphogenic protein
LOS	length of stay
ODI	Oswestry disability index
VAS	visual analog scale
TK	thoracic kyphosis
SS	sacral slope
PI	pelvic incidence
SL	segmental lordosis
PT	pelvic tilt
LL	lumbar lordosis
SVA	sagittal vertical axis
DH	disk height
FH	foraminal height
CCI	Charlson comorbidity index
