# Supplementary material for: The Limited Evidence Base for Multilevel Lumbar Interbody Fusion and Its Consequences for Clinical Conclusions: A Systematic Review
Source: J Clin Med. 2026 Mar 17;15(6):2289. doi: 10.3390/jcm15062289 (PMC13026321; doi:10.3390/jcm15062289)
Supplement: Supplementary file 1 [file jcm-15-02289-s001.zip › JCM_Multilevel_Table_S1.pdf]

Table S1. Full Literature Search Strategy

Keywords	(("XLIF"[All Fields] OR (("extreme"[All Fields] OR "extremely"[All Fields] OR "extremes"[All Fields]) AND ("lumbarised"[All Fields] OR "lumbarization"[All Fields] OR "lumbarized"[All Fields] OR "lumbars"[All Fields] OR "lumbosacral region"[MeSH Terms] OR ("lumbosacral"[All Fields] AND "region"[All Fields]) OR "lumbosacral region"[All Fields] OR "lumbar"[All Fields]) AND "Interbody"[All Fields] AND ("fusion"[All Fields] OR "fusions"[All Fields])) OR (("extreme"[All Fields] OR "extremely"[All Fields] OR "extremes"[All Fields]) AND ("functional laterality"[MeSH Terms] OR ("functional"[All Fields] AND "laterality"[All Fields]) OR "functional laterality"[All Fields] OR "laterality"[All Fields] OR "lateral"[All Fields] OR "lateralisation"[All Fields] OR "lateralisations"[All Fields] OR "lateralise"[All Fields] OR "lateralised"[All Fields] OR "lateralises"[All Fields] OR "lateralising"[All Fields] OR "lateralities"[All Fields] OR "lateralization"[All Fields] OR "lateralizations"[All Fields] OR "lateralize"[All Fields] OR "lateralized"[All Fields] OR "lateralizes"[All Fields] OR "lateralizing"[All Fields] OR "laterally"[All Fields] OR "laterals"[All Fields]) AND "Interbody"[All Fields] AND ("fusion"[All Fields] OR "fusions"[All Fields])) OR "DLIF"[All Fields] OR (("direct"[All Fields] OR "directed"[All Fields] OR "directing"[All Fields] OR "direction compound"[Supplementary Concept] OR "direction compound"[All Fields] OR "direction"[All Fields] OR "directional"[All Fields] OR "directions"[All Fields] OR "directivities"[All Fields] OR "directivity"[All Fields] OR "directs"[All Fields]) AND ("lumbarised"[All Fields] OR "lumbarization"[All Fields] OR "lumbarized"[All Fields] OR "lumbars"[All Fields] OR "lumbosacral region"[MeSH Terms] OR ("lumbosacral"[All Fields] AND "region"[All Fields]) OR "lumbosacral region"[All Fields] OR "lumbar"[All Fields]) AND "Interbody"[All Fields] AND ("fusion"[All Fields] OR "fusions"[All Fields])) OR (("direct"[All Fields] OR "directed"[All Fields] OR "directing"[All Fields] OR "direction compound"[Supplementary Concept] OR "direction compound"[All Fields] OR "direction"[All Fields] OR "directional"[All Fields] OR "directions"[All Fields] OR "directivities"[All Fields] OR "directivity"[All Fields] OR "directs"[All Fields]) AND ("functional laterality"[MeSH Terms] OR ("functional"[All Fields] AND "laterality"[All Fields]) OR "functional laterality"[All Fields] OR "laterality"[All Fields] OR "lateral"[All Fields] OR "lateralisation"[All Fields] OR "lateralisations"[All Fields] OR "lateralise"[All Fields] OR "lateralised"[All Fields] OR "lateralises"[All Fields] OR "lateralising"[All Fields] OR "lateralities"[All Fields] OR "lateralization"[All Fields] OR "lateralizations"[All Fields] OR "lateralize"[All Fields] OR "lateralized"[All Fields] OR "lateralizes"[All Fields] OR "lateralizing"[All Fields] OR "laterally"[All Fields] OR "laterals"[All Fields]) AND "Interbody"[All Fields] AND ("fusion"[All Fields] OR "fusions"[All Fields])) OR (("direct"[All Fields] OR "directed"[All Fields] OR "directing"[All Fields] OR "direction compound"[Supplementary Concept] OR "direction compound"[All Fields] OR "direction"[All Fields] OR "directional"[All Fields] OR "directions"[All Fields] OR "directivities"[All Fields] OR "directivity"[All Fields] OR "directs"[All Fields]) AND ("functional laterality"[MeSH Terms] OR ("functional"[All Fields] AND "laterality"[All Fields]) OR "functional laterality"[All Fields] OR "laterality"[All Fields] OR "lateral"[All Fields] OR "lateralisation"[All Fields] OR "lateralisations"[All Fields] OR "lateralise"[All Fields] OR "lateralised"[All Fields] OR "lateralises"[All Fields] OR "lateralising"[All Fields] OR "lateralities"[All Fields] OR "lateralization"[All Fields] OR "lateralizations"[All Fields] OR "lateralize"[All Fields] OR "lateralized"[All Fields] OR "lateralizes"[All Fields] OR "lateralizing"[All Fields] OR "laterally"[All Fields] OR "laterals"[All Fields]) AND ("lumbarised"[All Fields] OR "lumbarization"[All Fields] OR "lumbarized"[All Fields] OR "lumbars"[All Fields] OR "lumbosacral region"[MeSH Terms] OR ("lumbosacral"[All Fields] AND "region"[All Fields]) OR "lumbosacral region"[All Fields] OR "lumbar"[All Fields]) AND "Interbody"[All Fields] AND ("fusion"[All Fields] OR "fusions"[All Fields])) OR "LLIF"[All Fields] OR (("functional laterality"[MeSH Terms] OR ("functional"[All Fields] AND "laterality"[All Fields]) OR "functional laterality"[All Fields] OR "laterality"[All Fields] OR "lateral"[All Fields] OR "lateralisation"[All Fields] OR "lateralisations"[All Fields] OR "lateralise"[All Fields] OR "lateralised"[All Fields] OR "lateralises"[All Fields] OR "lateralising"[All Fields] OR "lateralities"[All Fields] OR "lateralization"[All Fields] OR "lateralizations"[All Fields] OR "lateralize"[All Fields] OR "lateralized"[All Fields] OR "lateralizes"[All Fields] OR "lateralizing"[All Fields] OR "laterally"[All Fields] OR "laterals"[All Fields]) AND ("lumbarised"[All Fields] OR "lumbarization"[All Fields] OR "lumbarized"[All Fields] OR "lumbars"[All Fields] OR "lumbosacral region"[MeSH Terms] OR ("lumbosacral"[All Fields] AND "region"[All Fields]) OR "lumbosacral region"[All Fields] OR "lumbar"[All Fields]) AND "Interbody"[All Fields] AND ("fusion"[All Fields] OR "fusions"[All Fields])) OR "OLIF"[All Fields] OR (("oblique"[All Fields] OR "obliquely"[All Fields] OR "obliqueness"[All Fields] OR "obliques"[All Fields] OR "obliquities"[All Fields] OR "obliquity"[All Fields]) AND ("functional laterality"[MeSH Terms] OR ("functional"[All Fields] AND "laterality"[All Fields]) OR "functional laterality"[All Fields] OR "laterality"[All Fields] OR "lateral"[All Fields] OR "lateralisation"[All Fields] OR "lateralisations"[All Fields] OR "lateralise"[All Fields] OR "lateralised"[All Fields] OR "lateralises"[All Fields] OR "lateralising"[All Fields] OR "lateralities"[All Fields] OR "lateralization"[All Fields] OR "lateralizations"[All Fields] OR "lateralize"[All Fields] OR "lateralized"[All Fields] OR "lateralizes"[All Fields] OR "lateralizing"[All Fields] OR "laterally"[All Fields] OR "laterals"[All Fields]) AND "Interbody"[All Fields] AND ("fusion"[All Fields] OR "fusions"[All Fields])) OR (("oblique"[All Fields] OR "obliquely"[All Fields] OR "obliqueness"[All Fields] OR "obliques"[All Fields] OR "obliquities"[All

	Fields] OR "obliquity"[All Fields]) AND ("lumbarised"[All Fields] OR "lumbarization"[All Fields] OR "lumbarized"[All Fields] OR "lumbars"[All Fields] OR "lumbosacral region"[MeSH Terms] OR ("lumbosacral"[All Fields] AND "region"[All Fields]) OR "lumbosacral region"[All Fields] OR "lumbar"[All Fields]) AND "Interbody"[All Fields] AND ("fusion"[All Fields] OR "fusions"[All Fields])) OR (("oblique"[All Fields] OR "obliquely"[All Fields] OR "obliqueness"[All Fields] OR "obliques"[All Fields] OR "obliquities"[All Fields] OR "obliquity"[All Fields]) AND ("functional laterality"[MeSH Terms] OR ("functional"[All Fields] AND "laterality"[All Fields]) OR "functional laterality"[All Fields] OR "laterality"[All Fields] OR "lateral"[All Fields] OR "lateralisation"[All Fields] OR "lateralisations"[All Fields] OR "lateralise"[All Fields] OR "lateralised"[All Fields] OR "lateralises"[All Fields] OR "lateralising"[All Fields] OR "lateralities"[All Fields] OR "lateralization"[All Fields] OR "lateralizations"[All Fields] OR "lateralize"[All Fields] OR "lateralized"[All Fields] OR "lateralizes"[All Fields] OR "lateralizing"[All Fields] OR "laterally"[All Fields] OR "laterals"[All Fields]) AND ("lumbarised"[All Fields] OR "lumbarization"[All Fields] OR "lumbarized"[All Fields] OR "lumbars"[All Fields] OR "lumbosacral region"[MeSH Terms] OR ("lumbosacral"[All Fields] AND "region"[All Fields]) OR "lumbosacral region"[All Fields] OR "lumbar"[All Fields]) AND "Interbody"[All Fields] AND ("fusion"[All Fields] OR "fusions"[All Fields])) OR "TLIF"[All Fields] OR ("Transforaminal"[All Fields] AND ("lumbarised"[All Fields] OR "lumbarization"[All Fields] OR "lumbarized"[All Fields] OR "lumbars"[All Fields] OR "lumbosacral region"[MeSH Terms] OR ("lumbosacral"[All Fields] AND "region"[All Fields]) OR "lumbosacral region"[All Fields] OR "lumbar"[All Fields]) AND "Interbody"[All Fields] AND ("fusion"[All Fields] OR "fusions"[All Fields])) OR "PLIF"[All Fields] OR (("posterior"[All Fields] OR "posteriors"[All Fields]) AND ("lumbarised"[All Fields] OR "lumbarization"[All Fields] OR "lumbarized"[All Fields] OR "lumbars"[All Fields] OR "lumbosacral region"[MeSH Terms] OR ("lumbosacral"[All Fields] AND "region"[All Fields]) OR "lumbosacral region"[All Fields] OR "lumbar"[All Fields]) AND "Interbody"[All Fields] AND ("fusion"[All Fields] OR "fusions"[All Fields]))) AND ("multilevel"[All Fields] OR "multilevels"[All Fields] OR "multi-level"[All Fields] OR ("two"[All Fields] AND ("level"[All Fields] OR "levels"[All Fields])) OR ("2"[All Fields] AND ("level"[All Fields] OR "levels"[All Fields])) OR "2-level"[All Fields] OR "two-level"[All Fields])) NOT ("systematic review"[Publication Type] OR "systematic reviews as topic"[MeSH Terms] OR "systematic review"[All Fields] OR ("systematic review"[Publication Type] OR "systematic reviews as topic"[MeSH Terms] OR "systematic review"[All Fields]) OR ("meta analysis"[Publication Type] OR "meta analysis as topic"[MeSH Terms] OR "meta analysis"[All Fields]) OR ("meta analysis"[Publication Type] OR "meta analysis as topic"[MeSH Terms] OR "meta analysis"[All Fields]))
