# Supplementary material for: The Limited Evidence Base for Multilevel Lumbar Interbody Fusion and Its Consequences for Clinical Conclusions: A Systematic Review
Source: J Clin Med. 2026 Mar 17;15(6):2289. doi: 10.3390/jcm15062289 (PMC13026321; doi:10.3390/jcm15062289)
Supplement: Supplementary file 1 [file jcm-15-02289-s001.zip › JCM_Multilevel_Table_S2.pdf]

Table S2. Individual study indications, surgical details, author conclusions, and limitations

Study	LIF Procedure	Diagnosis or Indication for surgery	Type of Posterior Instrumentation	Bilateral or Unilateral Posterior Instrumentation	Graft Information	Conclusions	Limitations
Ahmadian et al. 2015	LLIF	Degenerative Disc Disease 10<br>Spondylolisthesis 2<br>Adult Degenerative Scoliosis 9	No posterior instrumentation used	NA	allograft + BMP	Stand alone lateral transpsoas interbody fusion has a role for single and multilevel disease showing improvements in clinical outcomes	Retrospective case series cohort with 12 month outcomes
Aono et al. 2018	PLIF	2-level degenerative spondylolisthesis 43<br>Degenerative spondylolisthesis with adjacent foraminal stenosis 4<br>Degenerative and isthmic spondylolisthesis 1	Pedicle Screw fixation	Bilateral	autograft from excised lamina and spinous process	Surgical outcomes of 2-level PLIF for degenerative lumbar diseases are satisfactory. However, we found that the fusion rate was 85%, which was inferior to that of single-level PLIF	Retrospectice case series, did not use multiplanar reconstruction CT in extension position possibly overestimating fusion rate
Chong et al. 2024	LLIF<br>TLIF	LLIF: Spondylolisthesis 11, Degenerative Scoliosis 16<br>TLIF: Spondylolisthesis 10, Degenerative Scoliosis 21	LLIF: Pedicle Screw Fixation and Coroent Cage<br>TLIF: Pedicle Screw Fixation and	Bilateral	LLIF: One half each of autogenous iliac bone graft and synthetic bone graft (collagen hybrid)<br>TLIF: deminerali	The superior radiological outcomes demonstrated by 2-level trans-psoas LLIF did not translate into any difference in clinical outcomes compared to 2-level TLIF at the 2-years	Small sample size introducing the possibility of type 2 error, retrospective introducing bias from surgeon preference, relatively short follow up

			Capstone Cage		zed bone matrix and local autograft bone obtained from unilateral total facetec tomy		
Claus et al. 2021	TLIF	Disc Herniation 2067<br>Recurrent Disc Herniation 127<br>Spinal Stenosis 2894<br>Spondylolisthesis 2047<br>Adjacent Segment Disease 466<br>Revision of Hardware 376<br>Other 174	NR	NR	NR	This analysis of a large, prospective, multicenter registry demonstrated that older age was not independently associated with increased risk of complications or worsening clinical outcomes following multilevel transforaminal lumbar interbody fusion	Limited to data collected by the MSSIC administers, retrospective bias, predictors in multivariate analysis that demonstrated an increased odds ratio without a likely clinically meaningful risk, missing data for PROS
Couture et al. 2004	PLIF	indications for fusion included plain x-ray films showing instability and magnetic resonance images demonstrating degenerative changes, central or foraminal stenosis, and disc collapse	pedicle screw fixation	Bilateral	HYDROSORB devices packed with morcellized autograft bone harvested from the elements	Our experience treating this small series of patients demonstrates that HYDROSORB bioabsorbable devices may be used to achieve satisfactory fusion rates and outcomes when used for PLIF procedures	Small cohort documenting an early experience with a novel PLIF technique, does not include outside independent review of radiographs

					of decom pression		
Du et al. 2019	TLIF	double-level spondylolytic spondylo listhesis 38	pedicle screw fixation and PEEK Cage	Bilateral	autograft	Posterior L4–S1 instrumented TLIF can reduce spondy lolisthesis, restore the sagittal lumbosacral alignment, and improve the clinical outcomes of patients with con tiguous double-level lumbar spondylolisthesis	This study did not compare the effectiveness of TLIF with other interbody fusion techniques, and the surgical advantages of TLIF still need to be further investigated by multi center, long-term follow-up
Ferraro et al. 2023	TLIF	lumbar back pain resistant to conservative treatment for more than 6 months with or without leg pain. Such patients were also afflicted with grade III or IV discopathy, instability, or foraminal post laminectomy stenosis and/or grade I–II degenerative spondylolisthesis or low-grade isthmic spondylolisthesis	pedicle screw fixation and TM Ardis Cage	Bilateral	autologous bone chips from the decompres sion area on the side of every cage	Our modified single-portal TLIF technique with a tan talum cage is a valid surgical solution in patients with disc pathologies with or without symptoms of radicu lar/foraminal stenosis and/or non-severe hypolordosis	Small patient sample limits the extent to which the findings can be generalized. Secondly, the study lacks a comparative analysis with other commonly used materials like PEEK or porous titanium.

Fujimori et al. 2020	PLIF (Lumbar)<br>PLIF (Lumbosacral)	Lumbar: Spondylolisthesis 44, Foraminal stenosis 3, Spondylolisthesis and foraminal stenosis 1<br>Lumbosacral: Spondylolisthesis 13, Foraminal stenosis 9, Spondylolisthesis and foraminal stenosis 3	pedicle screw fixation and interbody cage	Bilateral	Bone grafts were harvested from the resected local bones	2-level PLIF at the lumbar segments demonstrated a high fusion rate and improvements in pain	First, the present study was a retrospective design and patients were not randomized. Second, patients were not blinded to the result of fusion status
Gu et al. 2014	TLIF (MIS)<br>TLIF (Open)	MIS: Symptomatic degenerative disc disease 15, Symptomatic two-level lumbar stenosis 18, Symptomatic lumbar stenosis with segmental instability 11<br>Open: Symptomatic degenerative disc disease 11, Symptomatic two-level lumbar stenosis 14, Symptomatic lumbar stenosis with segmental instability 13	pedicle screw fixation and a single PEEK cage	Bilateral	autologous bone was inserted obliquely across the disc space	MITLIF was superior to the traditional open TLIF in the management of two-level degenerative lumbar diseases	The sample size was small and the follow-up periods were short.
Gu et al. 2015	TLIF (Unilateral)<br>TLIF (Bilateral)	Unilateral: Two-level lumbar stenosis with lumbar instability 17, One-level spondylolisthesis (grade I) and adjacent spinal stenosis 18<br>Bilateral: Two-level lumbar stenosis with lumbar instability 18, One-level spondylolisthesis (grade	pedicle screw fixation and PEEK cage	Unilateral<br>Bilateral	autologous bone was inserted obliquely across the disc space	Unilateral instrumentation after two-level MITLIF provides similar clinical and radiological outcomes to bilateral fixation in two-level degenerative lumbar diseases	The sample size was small and the follow-up periods were short. Second, the error of radiological assessment was inevitable

		I) and adjacent spinal stenosis 21					
Guppy et al. 2021	PLIF	spondylolisthesis or stenosis undergoing primary single or multilevel PLFs and PLIFs	No posterior instrumen tation used	NA	BMP usage in 47.5% of patients	Using operative nonunions as an outcome measure,we found in a large cohort of patients from a prospective spine registry with >4 years of follow-up that there was no difference in operative nonunions between PLFs and PLIFs	Many studies using registry data, the lack of randomization could have introduced different kinds of biases. There is some heterogeneity present in our data with respected to types of grafts used, be it allograft, allograft, or bone morphogenic protein (BMP)
Hacken burg et al. 2005	TLIF	isthmic spondylolistheses and degenerative disorders of the lumbar spine	pedicle screw fixation and curved DePuy AG Cage	Bilateral	the anterior part of the disc space is packed with autologous bone chips taken from the iliac crest through the same skin incision	The clinical outcome of the TLIF appears to be com parable with that reported in the literature for interbody fusions using the PLIF or ALIF technique. The post operative improvement of both the ODI and the VAS values were significant at follow-up	NR

Hioki et al. 2005	PLIF	Degenerative spondylolisthesis: 14<br>Spinal canal stenosis: 9<br>Intervertebral disc herniation: 4<br>Degenerative Scoliosis: 1	pedicle screw fixation	NR	autogenous bone graft from the lamina and spinous process	We reported our retrospective study of the clinical outcomes of double-level PLIF for low back degenerative disorders. Double-level PLIF provided satisfactory results by preserving the lordosis of the lumbar spine, although the surgical invasion was not minimal	The number of patients was relatively small and there was no control group. Moreover, the postoperative follow-up period might be too short to assess the risk of the adjacent disc levels becoming degenerated
Kalinin et al. 2020	TLIF (ARP)<br>TLIF (Traditional)	the presence of lower back pain and radicular clinical symptoms due to degenerative disease of the lumbar spine, the involvement of two adjacent vertebral segments, and the absence of improvement after conservative treatment for 6–8 weeks	NR	NR	NR	In this study, the accelerated recovery program has shown its safety and high clinical efficacy for patients with degenerative diseases of the lumbar spine	A significant limitation of the present study is its single-center setting and small numbers of patients
Kim et al. 2011	PLIF	1) no previous lumbar surgery, 2) instability accompanying spinal stenosis or spondylolisthesis at more than two levels, 3) followed up for more than 12 months, and 4) no severe osteoporosis	percutaneous pedicle screw fixation and PEEK cage	Bilateral	local bone chips harvested from the lamina and facet joints were inserted at each level	The vertical axis and detachable screw extender system for multi-level interbody fusion makes rod manipulation easier to perform. This technique can reduce the size of the midline skin incision as well as the risks for iatrogenic muscle injury	NR

Kurra et al. 2018	TLIF	symptomatic lumbar spinal stenosis as well as a concomitant spinal deformity	pedicle screw fixation	Bilaeral	NR	Coronal curve correction rates were noted between 10% and 30%. Our particular study did see modest improvements in the deformity correction; however, the primary goal of the surgery was for the relief of leg pain.	The retrospective nature of the study and limitations in its small sample size
Lee et al. 2016	TLIF (MIS)<br>TLIF (Open)	MIS: Degenerative Disc Disease 3, Spinal Stenosis 14, Spondylolisthesis 10<br>Open: Degenerative Disc Disease 4, Spinal Stenosis 25, Spondylolisthesis 14	MIS: percutaneous pedicle screw fixation and single PEEK cage<br>Open: Pedicle Screw fixation and two PEEK cages	MIS: Bilateral<br>Open: Bilateral	MIS: autologous local bone<br>Open: autologous local bone	MIS and conventional TLIF produced similar clinical and radiologic outcomes in multilevel lumbar fusion. However, based on the differences in perioperative outcomes, MIS TLIF may be a better choice for 2-or 3-segment lumbar fusion compared with conventional TLIF.	Follow up of 1 year, a small number of patients, and no complete determination of fusion rates at 2 years
Li et al. 2024	TLIF (n-HA/PA66)<br>TLIF (PEEK)	n-HA/PA66: Spinal stenosis 21, recurrent lumbar disc herniation 3, lumbar spondylolisthesis 24<br>PEEK: Spinal stenosis 24, recurrent lumbar disc herniation 4, lumbar spondylolisthesis 20	n-HA/PA66: pedicle screw fixation and n-HA/PA66 cage<br>PEEK: pedicle screw	Bilateral	Autologous bone obtained from the decompression	Overall, our data suggest that the outcomes of n-HA/PA66 cage group are comparable to those of the PEEK cage group, with a similar high fusion rate and low cage subsidence rate as PEEK cages, except its lower rate of ASD occurrence.	The single-center nature and small sample size affects the representativeness of our sample with respect to the target population

			fixation and PEEK cage				
Li et al. 2018	PLIF	(1) neurogenic claudication or radicular leg pain with associated neurologic signs, (2) preoperative radiologic examination showing multilevel (consecutive 3-level or 4-level) LDD, and (3) no response toatleast 6monthsofconservativetreatment.Onlythepatientswho meet the 3 criteria at the same time were included	pedicle screw fixation	NR	morselized bone was inserted into the intervertebral space	A hybrid technique including 1-level interbody fusion and multilevel posterolateral fusion is recommended for patients with multilevel LDD	NR

Liu et al. 2016	TLIF (UPS)<br>TLIF (UPS FS)<br>TLIF (BPS)	UPS: Severe spinal stenosis with instability 12, Two-level spondylolisthesis or one-level spondylolistheis with adjacent spinal stenosis 6, One-level recurrent disc herniation with adjacent spinal stenosis 4<br>UPSFS: Severe spinal stenosis with instability 17, Two-level spondylolisthesis or one-level spondylolistheis with adjacent spinal stenosis 6, One-level recurrent disc herniation with adjacent spinal stenosis 5<br>BPS: Severe spinal stenosis with instability 17, Two-level spondylolisthesis or one-level spondylolistheis with adjacent spinal stenosis 12, One-level recurrent disc herniation with adjacent spinal stenosis 5	UPS: Pedicle screw fixation and single PEEK cage<br>UPSFS: Pedicle screw fixation, single PEEK cage, translaminar facet screw<br>BPS: Pedicle screw fixation and PEEK cages	UPS/UPSFS: Unilateral<br>BPS: Bilateral	autologous bone	UPS or UPSFS instrumented TLIF could achieve satisfactory mid-term clinical outcome comparable to BPS's, with less surgical time, less blood loss, and lower cost; UPS should be prudently performed for two-level cases in case of lower fusion rate, and cannulated screws should be replaced by stronger solid screws in UPSFS to reduce facet screw breakage	Firstly, it is a retrospective case-control study, which inevitably has selection and recall bias, despite the fact that we collected and analyzed the data meticulously. Secondly, the operations of three groups were performed by two surgeons independently, which would result in some individual difference.

Lu et al. 2015	PLIF (Group A, Solo) PLIF (Group B, DIAM)	lumbar spinal stenosis, disc degeneration, and spondylolisthesis, alone or in combination, involving 2 to 4 disc segments including the L5–S1 level	Group A: pedicle screw fixation and PEEK cage Group B: pedicle screw fixation and PEEK cage, silicon based interspinous device - DIAM	Bilateral	NR	Providing a dynamic transition zone with a DIAM implant placed immediately proximal to a multilevel PLIF construct was associated with a significant reduction in the occurrence of radiographic ASD, compared with PLIF alone	NR
Luan et al. 2022	TLIF	Degenerative spondylolisthesis 23 Isthmic spondylolisthesis 15	pedicle screw fixation and cage	Bilateral and Unilateral	Autologous bone particles	Whether it is L3/L4 double-level spondylolisthesis or L4/ L5 double-level spondylolisthesis, double-level TLIF can effectively relieve the symptoms of patients and improve the functional status of patients, while improving the sag ittal sequence of the spine and improving the quality of life of patients based on restoring spondylolisthesis	This study is a single-center institutional review, the sample size is small, there is still a lack of control of different surgical procedures, there are still defects

Mao et al. 2014	TLIF	two-level lumbar stenosis with instability 98	pedicle screw fixation and PEEK cage	Unilateral	autologous morselized bone from the facetectomy and laminectomy	This study's data suggest that bilateral decompression via a unilateral approach using unilateral pedicle screw fixation for two-level lumbar stenosis with instability is an effective and less invasive method than with bilateral constructs	Despite small case series with retrospective design and the absence of a control group, our study suggests that bilateral decompression via a unilateral approach using UPSF for two level lumbar stenosis with instability showed good mid-term clinical outcomes
Min et al. 2013	TLIF	spinal stenosis	percutaneous pedicle screw fixation and cage	Unilateral	bone chips obtained during laminectomy and either autogenous bone chips harvested from the pos terior superior iliac crest or synthetic hydroxyapatite	Regardless of the number of fused levels, minimally invasive transforaminal lumbar interbody fusion showed satisfactory clinical and radiological outcomes in patients with spinal stenosis, which suggests that it may be useful even for patients with multilevel spinal stenosis	first, because of a small number of the subjects , it was impossible to compare interbody height, segmental lordotic angle, and lumbar lordotic angle measurements under same conditions; second, as this study was designed to analyze comparatively MITLIF cases, it was not compared with conventional posterior fusion or posterior interbody fusion using conventional pedicle screws.

Nakashima et al. 2019	PLIF LLIF	patients who underwent PLIF and LLIF between 2009-2016	pedicle screw fixation and interbody cages	Bilateral	bone graft was packed around the cages	LLIF provided better segmental lordosis and LL than conventional standard PLIF in cases of short-level spinal fusion surgeries. In patients undergoing multilevel interbody fusion surgery, PT and TK were significantly altered after LLIF, and spinopelvic harmony was enhanced, even after short-level LLIF.	First, as this was a retrospective analysis, the angles of the utilized cages inevitably varied among patients who underwent PLIF. Second, the exclusion of a number of patients who underwent 2- or 3-level fusion affected the statistical power of these cases
Nourian et al. 2019	LLIF	Spondylolisthesis (grade 1 or 2), foraminal stenosis, adjacent segment disease, disc herniation, degenerative disc disease, central and lateral recess stenosis	pedicle screw fixation	Bilateral and Unilateral	recombinant human bone morphogenetic protein-2	We found successful interbody fusion using rhBMP-2 via LLIF in 92% of levels at 2 years. The fusion rate in this series is also similar to those found in other studies with rhBMP-2 applied through different surgical approaches.	Limitations of our study include a lack of a control group to compare the rate of fusion of rhBMP-2 with other sources of allograft, the inability to compare rhBMP-2 dosages per level in an accurate manner with other studies, the retrospective nature of the study results in a less reliable mechanism for the capture of complication data

Okuda et al. 2018	PLIF	degenerative spondylolisthesis, isthmic spondylolisthesis, lumbar spinal stenosis, degenerative lumbar scoliosis, lumbar disc herniation	pedicle screw fixation and carbon cages	Bilateral	autologous bone	From the present results, surgical procedures such as multisegment PLIF or lateral interbody fusion with pedicle screw fixation should be considered as the primary operation, if radiological foraminal stenosis at the adjacent fusion segment was observed preoperatively.	The present study had some limitations. First, there was a wide range of follow-up (2-21 years). The ASD rate and time period would change by the follow-up period, as mentioned above. However, the average follow-up period of 8.3 years was long compared with previous reports. Second, radiological risk factors for ASD such as spinopelvic parameters and preexisting disc degeneration were not investigated.
Ormond et al. 2012	TLIF	axial back pain with or without radiculopathy and lower extremity weakness	pedicle screw fixation and PEEK cage	Bilateral	Local bone graft from the lamina and demineralized bone matrix	PEEK rods in lumbar degenerative spine disease have A fusion rate of 89.3% and a reoperation rate of 19.1% in this small case series, and may not provide any improved benefit over instrumented fusions with all-metal implants	

Park et al. 2023	PLIF	spinal stenosis, isthmic or degenerative spondylolisthesis, and spinal instability	pedicle screw fixation and cages	Bilateral	laminectomized autologous bone	In this study, we demonstrated the successful bone fusion using low dose of E.BMP-2 (total 1mg) and local bone graft after a one-year follow-up period. These results indicate that the adjunctive use of E.BMP-2 with HA and b-TCP hydrogel can facilitate successful bone fusion in patients who may not have sufficient amounts of local auto graft or have poor bone quality	The incidence of osteoporosis in the subjects analyzed in this study was higher in females than in males, it was not sufficient to analyze the results of a relatively short observation period (12 months) to evaluate bone fusion after spinal fusion, and a comprehensive analysis of the cost-effectiveness of utilizing E.BMP-2 was not performed
Rouben et al. 2011	TLIF	foraminal lateral herniation of disc, central herniated disc, degenerative spondylolisthesis, degenerative disc disease, degenerative joint disease, central stenosis	pedicle screw fixation and PEEK cage	Bilateral and Unilateral	locally harvested auto logous bone and with rhBMP-2	Overall, MIS TLIF seems to be a safe and effective treatment. Future studies should evaluate the comparative benefit of MIS TLIF, Open TLIF, and simple decompression	This was not a randomized controlled trial with a comparator group, the authors of this study carried out MIS surgery as their primary standard of practice, and do not often carry out open surgery procedures, and as this was a single center study, no open surgical patients were available for comparison

Sakaura et al. 2018	PLIF	2-level degenerative lumbar spondylolisthesis	pedicle screw fixation and PEEK cages	Bilateral	local bone graft	Similar to our reported clinical and radiological outcomes in patients after single-level PLIF with CBT screw fixation for single-level DS compared with those after single-level PLIF using traditional PS fixation,15 2-level PLIF with CBT screw fixation for 2-level DS could be less invasive and provide surgical outcomes comparable to those of 2-level PLIF using traditional PS fixation	NR
Salehi et al. 2004	TLIF	two-level spondylolisthesis	pedicle screw fixation and cage	Bilateral	The previously harvested cancellous bone is packed inside the interbody space in the anterior and lateral portions as well as into the cages	This study demonstrates that TLIF can be performed safely and effectively to achieve circumferential fusion	Even though we did not compare our fusion rate with that of a control group in our institution, the results are similar to recent historical data with this type of procedure.

Song et al. 2015	PLIF (Autogenous bone chips, Group 1) PLIF (cage, Group 2)	two level symptomatic isthmic spondylolisthesis: 54	Group 1: pedicle screw fixation Group 2: PEEK Cage	Group 1: Bilateral Group 2: Unilateral	Group 1: Chips were compressed into the disk space to make it compact using bone taps Group 2: autologous lamina bone graft inserted obliquely across the disk space	In conclusion, both techniques could significantly improve pain and disability in patients with double-level isthmic spondylolisthesis. The efficacy of surgery on pain and disability improvement was similar in both groups	The retrospective nature of the review, the short-term follow-up and small sample size.
Song et al. 2017	PLIF	double-level isthmic spondylolisthesis: 32	pedicle screw fixation	Bilateral	the bone procured from the decompression was cleared of any soft tissue and chips were compressed into the disc space perform the bony fusion	Our treatment approach proved successful in this study. We therefore think that in treating double-level spondylolisthesis, a combination of decompression, anatomic reduction, and PLIF with autogenous bone chips seems to be a viable approach	Firstly, it was a retrospective with a short follow-up period; Secondly, a CT scan was not used for assessing fusion status because of expense and radiation protection

Takahashi et al. 2019	PLIF	2-level degenerative spondylolisthesis: 33	pedicle screw fixation	Bilateral	NR	The clinical outcomes of 2-level PLIF for 2-level DS limited at L3–4-5 appeared to be satisfactory. With respect to the relationships between clinical outcomes and sagittal spinopelvic parameters, ΔSL affected the clinical outcomes and a positive correlation was detected between ΔSL and ΔLL.	First, only a small number of patients were enrolled, and the study had a short duration of postoperative observation, this study was a retrospective review and postoperative radiological data were evaluated only at the time of the final follow-up, and only radiographic variables were analyzed as predictors of clinical outcome in this study
Talia et al. 2015	TLIF	spondylolisthesis, central canal stenosis, foraminal stenosis	pedicle screw fixation and peek cage	Bilateral	Bone graft was then packed into the disc spaces	The present study demonstrated that bilateral decompression as part of a TLIF procedure is a safe and effective alternative to the traditional TLIF, which utilises a unilateral window through the facet joint to access the disc space.	The limitations of our study include its retrospective nature, the relatively small sample size, and the lack of follow-up data greater than 12 months
Tsai et al. 2021	TLIF	L4 on L5 degenerative spondylolisthesis: 12	pedicle screw fixation and interbody cage	Bilateral	NR	Compared with traditional open spinal fusion surgery, MIS-TLIF provided an increased fusion rate and decreased pedicle screw loosening rate. It also afforded a	A limitation of our study is the small sample size

						more stable environment for in Situ and adjacent vertebral segments, thus preventing new VCFs	
Xi et al. 2020	OLIF (Nonobese) OLIF (Obese)	degenerative conditions of the lumbar spine (including deformity and spondylolisthesis)	NR	NR	NR	Although obesity generally does not increase approach related sequelae and complications during OLIF, when multilevel OLIF includes the L5–S1 level, patients have longer operative times and a higher incidence of transient, approach-related sequelae	First, this is a retrospective, single-institution study. Also, a minimum of 2 years of follow-up would have been ideal, but since this study focused on perioperative morbidity, most operative factors should have been captured with the follow-up in this study

Yang et al. 2014	PLIF	degenerative lumbar spinal canal stenosis, lumbar intervertebral disc herniation, spinal stenosis with disc herniation	NR	NR	NR	Implantation of Dynesys is less invasive than PLIF, such as less operation time and less blood loss and effectively improves the ODI and VAS for back and leg pain, stabilizes the unstable spine and protects the mobility of operated level, but no advantages on protection of ROM of adjacent segments and adjacent discs' degeneration have been observed in short term followup	The limitations of the study are firstly, although the mean followup time in Dynesys group is 2.22 ± 0.43 years and 2.17 ± 0.76 years in PLIF group, much longer followup is needed to determine the long term radiographic and clinical results of Dynesys. Secondly, small patient numbers may be the reason for the insignificant incidence of complications, ROM of adjacent segments and degenerative changes on adjacent discs. Thirdly, the data are from a single medical center, so further randomized controlled trial in multiple medical center should be conducted

Yoo et al. 2014	TLIF	patients with grade I/II of spondy lolytic or degenerative spondylolisthesis; patients who suffered from spinal stenosis or spinal disc herniation with degenerative segmental instability	percutaneous screw fixation and cage	Unilateral	hydroxyapatite and the bone harvested from an autogenous bone during laminectomy	Therefore, unilateral MITLIF can be considered to be an effective surgical method to minimize lumbar muscle damage, even at multiple levels	The number of cases was small, and this was a retrospective study. Factors such as general condition or preoperative neurology, which can affect clinical outcomes, were not considered
Yoon et al. 2023	OLIF TLIF	lumbar degenerative disorders	NR	NR	NR	The anterior approaches, including ALIF and OLIF, resulted in greater changes in ADH, DA, and FSL than TLIF. However, regarding global sagittal alignment, there were no significant differences in postoperative LL, PI-LL mismatch, or SVA between the groups, and the postoperative spinopelvic parameters were acceptable in all three groups. Although anterior approaches, including ALIF and OLIF, were superior to TLIF in terms of local radiographic parameters, TLIF could provide adequate global sagittal alignment, comparable to that of the anterior approaches	First, this study was retrospective and did not involve randomization, Second, the sample size was relatively small, particularly in the ALIF group. Because we shifted the principal anterior approach from ALIF to OLIF, the number of patients in the ALIF group was small. Third, we only included multi-level LIF surgeries and excluded single-level surgeries, although single-level LIFs vastly outnumber multi-level LIFs

Zhang et al. 2014	TLIF (Unilateral)<br>TLIF (Bilateral)	Unilateral: Spinal Stenosis 11, Symptomatic DDD 13, Spondylolisthesis 5, Failed Back Surgey 4<br>Bilateral: Spinal Stenosis 16, Symptomatic DDD 9, Spondylolisthesis 7, Failed Back Surgey 3	pedicle screw fixation and capstone cages	Unilateral<br>Bilateral	local<br>autograft	Unilateral instrumented TLIF with cages is an effective and safe method of treating two-level lumbar disease, which can take less operative time, reducing blood loss and hospital stay and implant costs	Considering different diagnoses in the two groups, the sample size in this study was relatively small, which may limit the comparability and outcomes. Furthermore, less rigid unilateral fixation is considered to reduce the possibility of adjacent segment pathology, but no radiological signs of adjacent segment pathology existed in either group
Zhang et al. 2018	PLIF	double-level spondylolisthesis: 24	pedicle screw fxation	bilateral	Internal fixation with autologous bone chip: 20<br>Cage and autogenous bone graft fixation: 4	We found that both surgical techniques could significantly improve pain and disability in patients with double-level lumbar spondylolisthesis and achieve good mid-term prognosis	We adopt dynamic lateral flexion and extension X-ray films to evaluate the fusion state, which is widely used. However, CT is more advantageous in evaluating the imaging diagnosis of spinal fusion. Because of the low incidence rate, the number of single centre samples is small and the conclusions are limited

Zhang et al. 2022	TLIF (MIS)<br>TLIF (Open)	spondylolisthesis, disc herniation with instability or spinal canal stenosis	pedicle screw fixation	Unilateral<br>Bilateral	PEEK cage with autologous bone	The contralateral bridge Freehand MIPS combined with unilateral MIS-TLIF (smile-face surgery) has advantages over open TLIF including smaller aggression, less blood loss, and lower cost, indicating that it is a good choice of treatment for multi-segmental LDDs	Smile-face surgery is a technically demanding procedure that has a learning curve, this study was a retrospective analysis, so there might be some bias in the selection of cases, normal physiological loads were applied to the model without destructive or excessive loads in the finite element analysis
Zhao et al. 2018	TLIF (Unilateral Incision)<br>TLIF (Bilateral Incision)	two-level lumbar stenosis, with or without grade I degenerative or isthmic lumbar spondylolisthesis	pedicle screw fixation (open and percutaneous) and PEEK cage	Bilateral	autologous bone	In conclusion, based on the bilateral decompression via unilateral approach technique, MIS-TLIF with bilateral short decompression incision could achieve comparable clinical results and reduce radiation exposure, shorten operative time, and decrease blood loss	Firstly, the sample size of our study was relatively small and the follow-up period was short, which might lower the reliability of the results. Secondly, the grouping was not prospectively randomized and some details of the treatment were unavailable, which was an inherent shortcoming of the retrospective study
