# Supplementary material for: The Limited Evidence Base for Multilevel Lumbar Interbody Fusion and Its Consequences for Clinical Conclusions: A Systematic Review
Source: J Clin Med. 2026 Mar 17;15(6):2289. doi: 10.3390/jcm15062289 (PMC13026321; doi:10.3390/jcm15062289)
Supplement: Supplementary file 1 [file jcm-15-02289-s001.zip › JCM_Multilevel_Table_S3.pdf]

Table S3. Pooled Complication Events and Weighted Rates

Complication Category	TLIF (N)	TLIF Weighted Rate, % (SD)	PLIF (N)	PLIF Weighted Rate, % (SD)	LLIF (N)	LLIF Weighted Rate, % (SD)	OLIF (N)	OLIF Weighted Rate, % (SD)
Total Patients	4104	-	949	-	45	-	137	-
Total Complications	1019	24.83% (10.74)	132	13.91% (21.91)	32	71.11% (13.05)	21	15.33% (10.07)
Revision/Return to OR	14	4.70% (12.25)	23	3.25% (7.38)	2	4.76%	0	0%
Dural Tear	16	2.91% (1.32)	11	4.04% (3.36)	0	0%	1	1.67%
Adjacent Segment Disease (ASD)	36	15.39% (11.14)	51	19.39% (14.66)	6	25%	0	0%
Pseudoarthrosis	1	1.89%	0	0%	0	0%	0	0%
Adjacent Disc Herniation	0	0%	2	4.17%	0	0%	0	0%
Rod Fracture/Hardware Failure	10	2.61% (3.0)	4	2.15% (1.60)	1	4.16%	0	0%
Deep Wound Infection	7	1.87% (1.36)	9	6.77% (4.24)	0	0%	1	1.67%

Superficial Wound Infection	91	2.62% (1.62)	2	2.70% (0.53)	0	0%	0	0%
Symptomatic Hematoma	40	1.26% (0.45)	6	3.45% (1.05)	0	0%	0	0%
Delayed Wound Healing	80	2.42% (0.48)	1	5%	0	0%	1	1.67%
Myocardial Infarction	20	0.64% (5.46)	0	0%	0	0%	1	1.67%
Pulmonary Embolism	24	0.77%	1	5.26%	1	4.76%	0	0%
Deep Vein Thrombosis	54	1.64% (1.12)	0	0%	0	0%	0	0%
Neurological Complication	19	0.56% (5.5)	11	9.74% (3.43)	15	33.33% (12.63)	0	0%
Psoas Weakness	0	0%	0	0%	9	42.86%	0	0%
UTI (Urinary Tract Infection)	142	4.16% (1.19)	0	0%	0	0%	0	0%
Other	472	13.93% (6.42)	22	7.33% (8.57)	4	8.89% (5.47)	10	16.67%
