# Supplementary material for: The Limited Evidence Base for Multilevel Lumbar Interbody Fusion and Its Consequences for Clinical Conclusions: A Systematic Review
Source: J Clin Med. 2026 Mar 17;15(6):2289. doi: 10.3390/jcm15062289 (PMC13026321; doi:10.3390/jcm15062289)
Supplement: Supplementary file 1 [file jcm-15-02289-s001.zip › JCM_Multilevel_Table_S4.pdf]

Table S4: Complications Reported as Other

Study	LIF Procedure	Mean Follow Up ± SD(range)[95% CI]{IQR}, months	# of pts	Other, explain
Ahmadian et al. 2015	LLIF	14.57±4.86(12-24)	21	1 Urinary retention
Aono et al. 2018	PLIF	57.6(24-132)	48	3 Adjacent compression fracture
Chong et al. 2024	LLIF<br>TLIF	24	LLIF: 24<br>TLIF: 29	LLIF: 3, atelectasis, gouty flare, delayed emergence from anesthesia<br>TLIF: 2, vertigo, hemoptysis
Claus et al. 2021	TLIF	24	3120 Total<br>3117 Complication analysis<br>94 for PRO Analysis	9 Mortality<br>321 Urinary Retention<br>68 Ileus<br>57 CSF Leak
Couture et al. 2004	PLIF	26.1(17-32)	12	
Du et al. 2019	TLIF	34.2±11.8(24–73)	38	
Ferraro et al. 2023	TLIF	30 (24–58)	14	
Fujimori et al. 2020	PLIF (Lumbar)<br>PLIF (Lumbosacral)	Lumbar: 25.45 ± 3.72<br>Lumbosacral: 24.95 ± 4.27	Lumbar: 48<br>Lumbosacral: 25	
Gu et al. 2014	TLIF (MIS)<br>TLIF (Open)	MIS: 20.6±4.5<br>Open: 20.0±3.3	MIS: 44<br>Open: 38	
Gu et al. 2015	TLIF (Unilateral)<br>TLIF (Bilateral)	Unilateral: 32.1±7.5<br>Bilateral: 31.7±8.0	Unilateral: 35<br>Bilateral: 39	
Guppy et al. 2021	PLIF	63.13±38.64	440	

Hackenburg et al. 2005	TLIF	46(36-64)	13	
Hioki et al. 2005	PLIF	43.2±20.4(24-89)	19	
Kalinin et al. 2020	TLIF (ARP)<br>TLIF (Traditional)	ARP: 17.33±7.8<br>Traditional: 19±8.58	ARP: 24<br>Traditional: 29	
Kim et al. 2011	PLIF	25.3±7.161(12-43)	42	2, medial penetration of the pedicle border without neurological deficits
Kurra et al. 2018	TLIF	60	12	
Lee et al. 2016	TLIF (MIS)<br>TLIF (Open)	12	MIS: 27<br>Open: 43	
Li et al. 2024	TLIF (n-HA/PA66)<br>TLIF (PEEK)	n-HA/PA66: 84.81± 9.60<br>PEEK: 87.48±9.40	n-HA/PA66: 48<br>PEEK: 48	PEEK: 1, CSF Leakage
Li et al. 2018	PLIF	48	46	3, CSF leakage
Liu et al. 2016	TLIF (UPS)<br>TLIF (UPSFS)<br>TLIF (BPS)	UPS: 46.4±6.0<br>UPSFS: 45.6±8.8<br>BPS: 46.4±5.5	UPS: 22<br>UPSFS: 28<br>BPS: 34	
Lu et al. 2015	PLIF (Group A, Solo)<br>PLIF (Group B, DIAM)	Group A: 41.5 ± 8.6(24-48)<br>Group B: 41.2 ± 7.2(24-48)	Group A: 42<br>Group B: 49	
Luan et al. 2022	TLIF	>12	36	
Mao et al. 2014	TLIF	36	98	
Min et al. 2013	TLIF	22.86±7.55(18-52)	58	
Nakashima et al. 2019	PLIF<br>LLIF	24	PLIF: 35<br>LLIF: 43	
Nourian et al. 2019	LLIF	20	22	
Okuda et al. 2018	PLIF	72	55	

Ormond et al. 2012	TLIF	33.28±16.1	7	
Park et al. 2023	PLIF	12	38	
Rouben et al. 2011	TLIF	>36 (36-60)	45	
Sakaura et al. 2018	PLIF	35.4 ± 11.4	20	
Salehi et al. 2004	TLIF	15.5±10.71	11	1, Pseudomeningocele
Song et al. 2015	PLIF (Autogenous bone chips, Group 1)<br>PLIF (cage, Group 2)	Group 1: 27.2<br>Group 2: 26.8	Group 1: 29<br>Group 2: 25	Group 1: 1, malpositioned screw requiring intraop repositioning
Song et al. 2017	PLIF	33.6 ± 11.76 (24-72)	32	2, malpositioned screw requiring intraop repositioning
Takahashi et al. 2019	PLIF	67.2±26.46 (24-132)	33	9 weakness
Talia et al. 2015	TLIF	12	12	
Tsai et al. 2021	TLIF	24	12	
Xi et al. 2020	OLIF (Nonobese)<br>OLIF (Obese)	Nonobese: 14.15 ± 9.79<br>Obese: 18.88 ± 9.97	Nonobese: 53<br>Obese: 24	
Yang et al. 2014	PLIF	26.04±9.12	45	2, aggravated back and leg pain
Yoo et al. 2014	TLIF	25.92	45	

Yoon et al. 2023	OLIF<br>TLIF	OLIF: 28.8±15.6<br>TLIF: 51.6±33.6	OLIF: 60<br>TLIF: 58	OLIF: 5, urinary disturbance<br>3 pulonary congestion<br>1 vascular injury<br>1 Ileus<br>TLIF: 6 Urinary Disturbance<br>2 Delirium<br>1 asthma attack
Zhang et al. 2014	TLIF (Unilateral)<br>TLIF (Bilateral)	Unilateral: 25.6±4.41(18–36)<br>Bilateral: 25.6±4.41(18–36)	Unilateral: 33<br>Bilateral: 35	
Zhang et al. 2018	PLIF	50.04±15 (24–78.96)	24	
Zhang et al. 2022	TLIF (MIS)<br>TLIF (Open)	MIS: 72.2±3.2<br>Open: 76.5±4.2	MIS: 45<br>Open: 37	Open: Severe low back pain 4
Zhao et al. 2018	TLIF (Unilateral Incision)<br>TLIF (Bilateral Incision)	Unilateral: 21.87±8.90<br>Bilateral: 24.56±10.08	Unilateral: 62<br>Bilateral: 67	
