# Supplementary material for: The Limited Evidence Base for Multilevel Lumbar Interbody Fusion and Its Consequences for Clinical Conclusions: A Systematic Review
Source: J Clin Med. 2026 Mar 17;15(6):2289. doi: 10.3390/jcm15062289 (PMC13026321; doi:10.3390/jcm15062289)
Supplement: Supplementary file 1 [file jcm-15-02289-s001.zip › JCM_Multilevel_Table_S5.pdf]

Table S5. Collected Patient Reported Outcome Measures

Study	LIF Procedure	Preoperative ODI mean ± SD(range)[95% CI]{IQR}	Postoperative ODI mean ± SD(range)[95% CI]{IQR}	Delta ODI	Preoperative VAS(leg) mean ± SD(range)[95% CI]{IQR}	Postoperative VAS(leg) mean ± SD(range)[95% CI]{IQR}	Delta VAS-L	Preoperative VAS(back) mean ± SD(range)[95% CI]{IQR}	Postoperative VAS(back) mean ± SD(range)[95% CI]{IQR}	Delta VAS-B	Preoperative JOA mean ± SD(range)[95% CI]{IQR}	Postoperative JOA mean ± SD(range)[95% CI]{IQR}	Preoperative JOABPEQ(lumbar function) mean ± SD(range)[95% CI]{IQR}	Postoperative JOABPEQ(lumbar function) mean ± SD(range)[95% CI]{IQR}	Preoperative SRS-22 mean ± SD(range)[95% CI]{IQR}	Postoperative SRS-22 mean ± SD(range)[95% CI]{IQR}	Preoperative EQ-5D mean ± SD(range)[95% CI]{IQR}	Postoperative EQ-5D mean ± SD(range)[95% CI]{IQR}	Preoperative SF-36 Physical mean ± SD(range)[95% CI]{IQR}	Postoperative SF-36 Physical mean ± SD(range)[95% CI]{IQR}	Preoperative PROMIS mean ± SD(range)[95% CI]{IQR}	Postoperative PROMIS mean ± SD(range)[95% CI]{IQR}	Preoperative PHQ-9 mean ± SD(range)[95% CI]{IQR}	Postoperative PHQ-9 mean ± SD(range)[95% CI]{IQR}	Preoperative PDQ mean ± SD(range)[95% CI]{IQR}	Postoperative PDQ mean ± SD(range)[95% CI]{IQR}	Patient Satisfaction, n(%)
Ahmadian et al. 2015	LLIF	50.55±12.38(32-74)	34.57±17.67(2-64)	-15.98±5.29				6.778±1.648(4-10)	3.920±2.545(0-10)	2.858±0.897																	
Aono et al. 2018	PLIF										12.1±2.7	23.8±2.8															
Chong et al. 2024	LLIF TLIF	LLIF: 49.9±17.3 TLIF: 50.01±19.3	LLIF: 24.5±25.9 TLIF: 16.2±16.6	LLIF: -25.4±8.6 TLIF: 33.81±2.7	LLIF: 5.61±4.03 TLIF: 5.58±3.49	LLIF: 2.2±4.1 TLIF: 0.8±2.4	LLIF: 3.41±0.07 TLIF: 4.78±1.09	LLIF: 6.52±3.13 TLIF: 5.7±3.2	LLIF: 2.3±3.6 TLIF: 1.3±2.7	LLIF: 4.22±0.47 TLIF: 4.4±0.5									LLIF: 36.4±26.4 TLIF: 38.85±30.2	LLIF: 62.2±26.9 TLIF: 65.8±26.7							
Claus et al. 2021	TLIF				6.8±2.7	2.5±3.0	4.3±0.3	7.4±2.3	3.9±2.9	3.5±0.6							0.51±0.21	0.72±0.21			34.6±5.1	40.7±8.1					
Couture et al. 2004	PLIF																										
Du et al. 2019	TLIF	56.3±21.3	31.7±16.4	24.6±4.9	8.1 ± 2.2	2.4 ± 1.6	5.7 ± 0.8	7.2 ± 3.5	2.9 ± 1.7	4.3 ± 1.8									32.6 ± 7.8	45.7 ± 6.3							

Ferraro et al. 2023	TLIF																										
Fujimori et al. 2020	PLIF (Lumbar)<br>PLIF (Lumbosacral)				Lumbar: 7.1<br>Lumbosacral: 6.6	Lumbar 2.3<br>Lumbosacral: 3.7	Lumbar 4.8<br>Lumbosacral: 2.9	Lumbar: 6.2<br>Lumbosacral: 6.1	Lumbar: 2.4<br>Lumbosacral: 3.8	Lumbar: 3.8<br>Lumbosacral: 2.3			Lumbar: 5.8<br>Lumbosacral: 5.1	Lumbar: 7.1<br>Lumbosacral: 7.3													
Gu et al. 2014	TLIF (MIS)<br>TLIF (Open)	MIS: 43.7±4.3<br>Open: 44.3±5.2	MIS: 16.5±2.0<br>Open: 15.9±1.9	MIS: 27.2±2.3<br>Open: 28.4±5.3	MIS: 7.6±0.9<br>Open: 7.7±0.9	MIS: 1.7±0.6<br>Open: 1.8±0.7	MIS: 5.9±0.3<br>Open: 5.9±0.2	MIS: 7.3±1.2<br>Open: 7.4±1.0	MIS: 1.9±0.7<br>Open: 1.8±0.6	MIS: 5.4±0.5<br>Open: 5.6±0.4																	
Gu et al. 2015	TLIF (Unilateral)<br>TLIF (Bilateral)	Unilateral: 44.51±5.03<br>Bilateral: 45.13±4.42	Unilateral: 17.10±1.48<br>Bilateral: 16.92±1.11	Unilateral: 27.41±3.55<br>Bilateral: 28.21±3.31	Unilateral: 7.91±0.85<br>Bilateral: 7.79±1.01	Unilateral: 1.97±0.62<br>Bilateral: 1.77±0.54	Unilateral: 5.94±0.23<br>Bilateral: 6.02±0.47	Unilateral: 7.31±1.05<br>Bilateral: 7.13±1.24	Unilateral: 1.91±0.51<br>Bilateral: 1.79±0.66	Unilateral: 5.4±0.54<br>Bilateral: 5.34±0.58																	
Guppy et al. 2021	PLIF																										
Hackenburg et al. 2005	TLIF	55.1±18.2	40±18.7	15.1±0.5				8.2±2.4	5.5±2.7	2.7±0.3																	
Hioki et al. 2005	PLIF										12.9±3.5	21.3±4.9															
Kalinin et al. 2020	TLIF (ARP)<br>TLIF (Traditional)																										
Kim et al. 2011	PLIF							7.5	2.9	4.6																	

Kurra et al. 2018	TLIF																	0.8±0.03						1.5±2.1		27±21	
Lee et al. 2016	TLIF (MIS) TLIF (Open)	MIS: 22.73±8.15 Open: 21.76±6.45	MIS: 5.73±3.45 Open: 4.30±2.83	MIS: 17.0±4.7 Open: 17.46±3.62				MIS: 6.85±1.66 Open: 5.79±1.5	MIS: 2.43±1.27 Open: 1.19±1.51	MIS: 4.42±0.39 Open: 4.6±0.01																	
Li et al. 2024	TLIF (n-HA/PA66) TLIF (PEEK)	n-HA/PA66: 54.29±8.41 PEEK: 54.79±7.81	n-HA/PA66: 11.83±2.89 PEEK: 12.63±4.78	n-HA/PA66: 42.46±5.52 PEEK: 42.16±3.03	n-HA/PA66: 6.65±0.98 PEEK: 6.40±0.94	n-HA/PA66: 1.63±0.89 PEEK: 1.81±0.64	n-HA/PA66: 5.02±0.09 PEEK: 4.59±0.3	n-HA/PA66: 6.60±1.05 PEEK: 6.65±1.00	n-HA/PA66: 2.00±1.05 PEEK: 2.02±0.79	n-HA/PA66: 4.60±0.0 PEEK: 4.63±0.21																	
Li et al. 2018	PLIF	48.7±18.2			7.4±1.6			6.8±1.4											28.8±11.7								
Liu et al. 2016	TLIF (UPS) TLIF (UPSFS) TLIF (BPS)	UPS: 46.5 ± 15.2 UPSFS: 48.0 ± 18.1 BPS: 47.3 ± 17.4	UPS: 6.5 ± 2.8 UPSFS: 5.2 ± 3.1 BPS: 5.6 ± 3.0	UPS: 40 ± 12.4 UPSFS: 42.8 ± 15.0 BPS: 41.7 ± 14.4	UPS: 7.8 ± 2.5 UPSFS: 7.7 ± 2.9 BPS: 7.5 ± 3.0	UPS: 1.5 ± 0.6 UPSFS: 1.2 ± 0.7 BPS: 0.9 ± 0.8	UPS: 6.3 ± 1.9 UPSFS: 6.5 ± 2.2 BPS: 6.6 ± 2.2	UPS: 6.5 ± 2.0 UPSFS: 7.0 ± 3.1 BPS: 6.8 ± 2.6	UPS: 2.2 ± 1.0 UPSFS: 2.3 ± 0.9 BPS: 2.5 ± 1.2	UPS: 4.3 ± 1.0 UPSFS: 4.7 ± 2.2 BPS: 4.3 ± 1.4	UPS: 11.2 ± 6.4 UPSFS: 9.9 ± 5.7 BPS: 10.8 ± 6.7	UPS: 28.5 ± 7.0 UPSFS: 27.9 ± 6.5 BPS: 28.0 ± 6.9															
Lu et al. 2015	PLIF (Group A, Solo) PLIF (Group B, DIAM)	Group A: 26.3 ± 3.4 Group B: 27.7 ± 3.8	Group A: 15.5 ± 4.2 Group B: 14.1 ± 3.9	Group A: 10.8 ± 0.8 Group B: 13.6 ± 0.1	Group A: 7.1 ± 1.4 Group B: 7.2 ± 1.3	Group A: 1.5 ± 2.2 Group B: 1.4 ± 2.3	Group A: 5.6 ± 0.8 Group B: 5.8 ± 1.0	Group A: 7.0 ± 1.2 Group B: 7.1 ± 1.4	Group A: 1.5 ± 2.1 Group B: 1.5 ± 2.4	Group A: 5.5 ± 0.9 Group B: 5.6 ± 1.0																	

Luan et al. 2022	TLIF	34.0 ± 7.5	18.9 ± 4.8	15.1 ± 2.7	5.2 ± 1.7	1.6 ± 1.1	3.6 ± 0.6	5.1 ± 1.7	2.7 ± 1.3	2.4 ± 0.4	14.9 ± 4.4	25.6 ± 2.3															
Mao et al. 2014	TLIF	51.01±13.22	5.21±3.83	45.8±9.39				7.02±2.34	0.67±0.18	6.35±2.16																	
Min et al. 2013	TLIF	26.22±3.65	9.65±3.05	16.57±0.6	7.8±0.83	1.12±0.2	6.68±0.63	6.48±1.22	1±0.48	5.48±0.74																	
Nakashima et al. 2019	PLIF LLIF										13.0±4.8																
Nourian et al. 2019	LLIF																										
Okuda et al. 2018	PLIF																										
Ormond et al. 2012	TLIF																										
Park et al. 2023	PLIF																										
Rouben et al. 2011	TLIF	73±12	27±19	46±7				7.7±1.3	2.6±2.1	5.1±0.8																	
Sakaura et al. 2018	PLIF										12.8 ± 4.2	20.4 ± 6.2*															

Salehi et al. 2004	TLIF																										
Song et al. 2015	PLIF (Autogenous bone chips, Group 1)<br>PLIF (cage, Group 2)	Group 1: 54 ± 21<br>Group 2: 60 ± 18.9	Group 1: 14.2 ± 12.6<br>Group 2: 12.6 ± 8.8	Group 1: 39.8 ± 8.4<br>Group 2: 47.4 ± 10.1	Group 1: 5.8 ± 3.1<br>Group 2: 6.1 ± 3.3	Group 1: 2.1 ± 2.2<br>Group 2: 1.8 ± 1.2	Group 1: 3.7 ± 0.9<br>Group 2: 4.3 ± 2.1	Group 1: 7.2 ± 3.4<br>Group 2: 7.0 ± 2.3	Group 1: 2.2 ± 2.1<br>Group 2: 1.9 ± 1.5	Group 1: 5.0 ± 1.3<br>Group 2: 5.1 ± 0.8																	
Song et al. 2017	PLIF							6.48	1.82	4.66	13.8 ± 3.1	25.6 ± 1.3															
Takahashi et al. 2019	PLIF										10.8± 4.5	19.6±7.4															
Talia et al. 2015	TLIF							8.2 ± 1.5 [7.2–9.1]	1.6 ± 0.87 [1.1–2.2]	6.6 ± 0.73																	
Tsai et al. 2021	TLIF																										
Xi et al. 2020	OLIF (Nonobese)<br>OLIF (Obese)																										
Yang et al. 2014	PLIF	72.82± 5.72	39.07± 2.65	33.75±3.07	7.26±0.76	3.56±0.38	3.70± 0.38	6.97±0.84	3.19±0.19	3.78± 0.65																	
Yoo et al. 2014	TLIF	43.26± 18.65	12.99± 9.25	30.27±9.4	7.55±2.52	0.85±1.01	6.7±1.51	5.94±2.74	1.82±1.47	4.12± 1.27																	

Yoon et al. 2023	OLIF<br>TLIF																										
Zhang et al. 2014	TLIF (Unilateral)<br>TLIF (Bilateral)	Unilateral: 42.4±16<br>Bilateral: 44.3±18.2	Unilateral: 18.8±3.2<br>Bilateral: 17.9±7.6	Unilateral: 23.6 ±12.8<br>Bilateral: 26.4 ±10.6	Unilateral: 7.4±2.8<br>Bilateral: 7.1±1.3	Unilateral: 1.9±1.4<br>Bilateral: 2.0±1.3	Unilateral: 5.5±1.4<br>Bilateral: 5.1±0.0	Unilateral: 7.8±0.8<br>Bilateral: 8.1±0.7	Unilateral: 2.1±0.8<br>Bilateral: 2.0±1.8	Unilateral: 5.7±0.0<br>Bilateral: 6.1±1.1									Unilateral: 16.7±6.2<br>Bilateral: 15.8±2.5	Unilateral: 47.2±15.2<br>Bilateral: 46.1±12.7							
Zhang et al. 2018	PLIF							8.88 ±1.36	2.25 ±1.26	6.63 ±0.1																	
Zhang et al. 2022	TLIF (MIS)<br>TLIF (Open)	MIS: 62.3±10.1<br>Open: 58.4±10.3	MIS: 4.8±2.4<br>Open: 5.4±2.9	MIS: 57.5 ±7.7<br>Open: 53.0 ±7.4	MIS: 7±0.924(5–9)<br>Open: 7±0.98 (5–9)	MIS: 1±0.231(0–1)<br>Open: 1±0.245(0–1)	MIS: 6±0.693<br>Open: 6±0.735	MIS: 6±1.16 (3–8)<br>Open: 6±1.47 (2–8)	MIS: 2±0.231(2–3)<br>Open: 2±0.49 (2–4)	MIS: 4±0.929<br>Open: 4±0.98																	
Zhao et al. 2018	TLIF (Unilateral Incision)<br>TLIF (Bilateral Incision)	Unilateral: 51.98 ± 12.69<br>Bilateral: 55.81 ± 13.74	Unilateral: 14.93 ± 5.92<br>Bilateral: 14.54 ± 6.53	Unilateral: 37.05 ± 6.77<br>Bilateral: 41.27 ± 7.21	Unilateral: 6.04 ± 0.47<br>Bilateral: 6.14 ± 0.57	Unilateral: 1.64 ± 0.23<br>Bilateral: 1.67 ± 0.28	Unilateral: 4.4 ± 0.24<br>Bilateral: 4.47 ± 0.29	Unilateral: 5.32 ± 0.56<br>Bilateral: 5.31 ± 0.61	Unilateral: 1.44 ± 0.34<br>Bilateral: 1.48 ± 0.33	Unilateral: 3.88± 0.22<br>Bilateral: 3.83± 0.28																	
