# Supplementary material for: The Limited Evidence Base for Multilevel Lumbar Interbody Fusion and Its Consequences for Clinical Conclusions: A Systematic Review
Source: J Clin Med. 2026 Mar 17;15(6):2289. doi: 10.3390/jcm15062289 (PMC13026321; doi:10.3390/jcm15062289)
Supplement: Supplementary file 1 [file jcm-15-02289-s001.zip › PRISMA_2020_checklist_Multilevel_LIF.pdf]

PRISMA 2020 Checklist

Section and Topic	Item #	Checklist item	Location where item is reported
**TITLE**			
Title	1	Identify the report as a systematic review.	**Title:** "The Limited Evidence Base for Multilevel Lumbar Interbody Fusion and its Consequences for Clinical Conclusions: A Systematic Review"
**ABSTRACT**			
Abstract	2	See the PRISMA 2020 for Abstracts checklist.	**Abstract:** The abstract has been structured with objectives, methods, results, and conclusions.
**INTRODUCTION**			
Rationale	3	Describe the rationale for the review in the context of existing knowledge.	**Introduction:** "This is limited to single-level procedures, and, to the author's knowledge, there is no prior study that has extensively reported on exclusively multilevel LIF procedures"
Objectives	4	Provide an explicit statement of the objective(s) or question(s) the review addresses.	**Abstract:** "This systematic review aimed to comprehensively review..."<br>**Introduction:** "Thus, this systematic review aims to thoroughly present..."
**METHODS**			
Eligibility criteria	5	Specify the inclusion and exclusion criteria for the review and how studies were grouped for the syntheses.	**Materials and Methods (Section 2.1):** "Inclusion criteria consisted of Level I-IV studies...", "Exclusion criteria included any non-full-text articles, biomechanical..."
Information sources	6	Specify all databases, registers, websites, organisations, reference lists and other sources searched or consulted to identify studies. Specify the date when each source was last searched or consulted.	**Materials and Methods (Section 2.1):** "A literature search was conducted by two authors using Pubmed, Embase, Web of Science, and Cochrane Central Register of Controlled Trials..."
Search strategy	7	Present the full search strategies for all databases, registers and websites, including any filters and limits used.	**Materials and Methods (Section 2.1):** "A literature search was conducted by two authors using Pubmed, Embase, Web of Science, and Cochrane Central Register of Controlled Trials...", "The literature search can be found in Table S1"
Selection process	8	Specify the methods used to decide whether a study met the inclusion criteria of the review, including how many reviewers screened each record and each report retrieved, whether they worked independently, and if applicable, details of automation tools used in the process.	**Materials and Methods (Section 2.1):** "A literature search was conducted by two authors using Pubmed, Embase, Web of Science, and Cochrane Central Register of Controlled Trials...", "Any conflicts were resolved by the senior author"
Data collection process	9	Specify the methods used to collect data from reports, including how many reviewers collected data from each report, whether they worked independently, any processes for obtaining or confirming data from study investigators, and if applicable, details of automation tools used in the process.	**Materials and Methods (Section 2.2):** "Data was extracted from the included studies by two authors and transferred to a Microsoft Excel..."
Data items	10a	List and define all outcomes for which data were sought. Specify whether all results that were compatible with each outcome domain in each study were sought (e.g. for all measures, time points, analyses), and if not, the methods used to decide which results to collect.	**Materials and Methods (Section 2.1):** "Inclusion criteria consisted of Level I-IV studies evaluating outcomes of lateral and posterior LIF of two or more levels, follow-up of at least one year, one or

Section and Topic	Item #	Checklist item	Location where item is reported
			more patient-reported outcome measures (PROM), radiographic measurements, postoperative complications, and fusion rates.”
	10b	List and define all other variables for which data were sought (e.g. participant and intervention characteristics, funding sources). Describe any assumptions made about any missing or unclear information.	**Materials and Methods (Section 2.2):** “The following data was extracted from each study: author name, publication year, level of evidence, demographics, operative details, radiographic outcomes, clinical outcomes, complications, conclusions and limitations.”
Study risk of bias assessment	11	Specify the methods used to assess risk of bias in the included studies, including details of the tool(s) used, how many reviewers assessed each study and whether they worked independently, and if applicable, details of automation tools used in the process.	**Materials and Methods (Section 2.3):** This section of the manuscript details all methodology for risk of bias assessment.
Effect measures	12	Specify for each outcome the effect measure(s) (e.g. risk ratio, mean difference) used in the synthesis or presentation of results.	Not explicitly described in the provided text.
Synthesis methods	13a	Describe the processes used to decide which studies were eligible for each synthesis (e.g. tabulating the study intervention characteristics and comparing against the planned groups for each synthesis (item #5)).	**Materials and Methods (Section 2.1):** “Inclusion criteria consisted of Level I-IV studies evaluating outcomes of lateral and posterior LIF of two or more levels, follow-up of at least one year, one or more patient-reported outcome measures (PROM), radiographic measurements, postoperative complications, and fusion rates.”
	13b	Describe any methods required to prepare the data for presentation or synthesis, such as handling of missing summary statistics, or data conversions.	**Materials and Methods (Section 2.2):** “All data was reported as mean with standard deviation and range, when possible. When data was presented without a standard deviation, the previously published approaches were used to derive an estimate [27, 28].”
	13c	Describe any methods used to tabulate or visually display results of individual studies and syntheses.	**Results:** Tables are utilized within the manuscript and supplemental file to visually display pooled weighted mean values.
	13d	Describe any methods used to synthesize results and provide a rationale for the choice(s). If meta-analysis was performed, describe the model(s), method(s) to identify the presence and extent of statistical heterogeneity, and software package(s) used.	**Materials and Methods (Section 2.2):** “All data was reported as mean with standard deviation and range, when possible.”, “All radiographic outcomes and PROMs were reported as change values between preoperative and postoperative measurements to assess the effect of the intervention. Due to significant heterogeneity in study design, a meta-analysis was not performed.”
	13e	Describe any methods used to explore possible causes of heterogeneity among study results (e.g. subgroup analysis, meta-regression).	**Abstract:** “Extensive subgroup analysis was completed when possible”<br>**Results:** Section 3.4 Comparative Outcomes explores numerous subgroup analyses
	13f	Describe any sensitivity analyses conducted to assess robustness of the synthesized results.	Not explicitly described in the provided text.

PRISMA 2020 Checklist

Section and Topic	Item #	Checklist item	Location where item is reported
Reporting bias assessment	14	Describe any methods used to assess risk of bias due to missing results in a synthesis (arising from reporting biases).	Not explicitly described in the provided text.
Certainty assessment	15	Describe any methods used to assess certainty (or confidence) in the body of evidence for an outcome.	Not explicitly described in the provided text.
**RESULTS**			
Study selection	16a	Describe the results of the search and selection process, from the number of records identified in the search to the number of studies included in the review, ideally using a flow diagram.	**Results (Section 3.1):** "The query of online databases identified 9294 studies. After initial screening, 1095 full texts were assessed for eligibility leading to an inclusion of 45 studies (Figure 1)."
	16b	Cite studies that might appear to meet the inclusion criteria, but which were excluded, and explain why they were excluded.	**Results (Section 3.1):** Figure 1 illustrates studies that were excluded in the review process.
Study characteristics	17	Cite each included study and present its characteristics.	**Results (Section 3.2):** This section details the study characteristics of each included study. "A detailed summary of reported study and patient characteristics can be found in Table 1."
Risk of bias in studies	18	Present assessments of risk of bias for each included study.	**Results (Section 3.5):** This section provides extensive assessment for risk of bias in nonrandomized and randomized study designs.
Results of individual studies	19	For all outcomes, present, for each study: (a) summary statistics for each group (where appropriate) and (b) an effect estimate and its precision (e.g. confidence/credible interval), ideally using structured tables or plots.	**Results (Section 3.3):** This section provides summary descriptive statistics for each outcome in the form of mean, standard deviation, and range when possible. Tables 2, 3, 4, and S3 provide additional summary statistics.
Results of syntheses	20a	For each synthesis, briefly summarise the characteristics and risk of bias among contributing studies.	Not explicitly completed for each synthesis. General comments are in the Limitations (Section 4.12) and Risk of Bias (Section 3.5) sections.
	20b	Present results of all statistical syntheses conducted. If meta-analysis was done, present for each the summary estimate and its precision (e.g. confidence/credible interval) and measures of statistical heterogeneity. If comparing groups, describe the direction of the effect.	**Results (Section 3.3):** This section provides summary descriptive statistics for each outcome in the form of mean, standard deviation, and range when possible. Tables 2, 3, 4, and S3 provide additional summary statistics. No formal statistical analysis was performed within this study.
	20c	Present results of all investigations of possible causes of heterogeneity among study results.	Not explicitly completed for each synthesis. General comments are in the Discussion (Section 4) and Limitations (Section 4.12) sections.
	20d	Present results of all sensitivity analyses conducted to assess the robustness of the synthesized results.	Not explicitly described in the provided text.
Reporting biases	21	Present assessments of risk of bias due to missing results (arising from reporting biases) for each synthesis assessed.	Not explicitly described in the provided text.
Certainty of evidence	22	Present assessments of certainty (or confidence) in the body of evidence for each outcome assessed.	Not explicitly described in the provided text.

PRISMA 2020 Checklist

Section and Topic	Item #	Checklist item	Location where item is reported
**DISCUSSION**			
Discussion	23a	Provide a general interpretation of the results in the context of other evidence.	**Discussion (Sections 4.1-4.11):** Interpretation of reported values is provided throughout the entire section.
	23b	Discuss any limitations of the evidence included in the review.	**Discussion (Section 4.12):** "Furthermore, inconsistent reporting and a publication bias within the literature increases the difficulty of reporting accurate values on LLIF and OLIF."
	23c	Discuss any limitations of the review processes used.	**Discussion (Section 4.12):** "Although this study utilized strict exclusion criteria, multilevel LIF literature is inherently heterogeneous. This limited our ability to quantitatively compares the procedures of LLIF, OLIF, TLIF, and PLIF."
	23d	Discuss implications of the results for practice, policy, and future research.	**Discussion (Section 4.12):** "Future studies can act to address this through...to reduce bias or confounding results."
**OTHER INFORMATION**			
Registration and protocol	24a	Provide registration information for the review, including register name and registration number, or state that the review was not registered.	Not explicitly described in the provided text.
	24b	Indicate where the review protocol can be accessed, or state that a protocol was not prepared.	Not explicitly described in the provided text.
	24c	Describe and explain any amendments to information provided at registration or in the protocol.	Not explicitly described in the provided text.
Support	25	Describe sources of financial or non-financial support for the review, and the role of the funders or sponsors in the review.	A statement on funding has been provided following the conclusion within the manuscript text.
Competing interests	26	Declare any competing interests of review authors.	A statement on any conflicts of interest has been provided following the conclusion within the manuscript text.
Availability of data, code and other materials	27	Report which of the following are publicly available and where they can be found: template data collection forms; data extracted from included studies; data used for all analyses; analytic code; any other materials used in the review.	A statement on data availability has been provided following the conclusion within the manuscript text.

From: Page MJ, McKenzie JE, Bossuyt PM, Boutron I, Hoffmann TC, Mulrow CD, et al. The PRISMA 2020 statement: an updated guideline for reporting systematic reviews. BMJ 2021;372:n71. doi: 10.1136/bmj.n71. This work is licensed under CC BY 4.0. To view a copy of this license, visit https://creativecommons.org/licenses/by/4.0/
